# Plasma‐Modified Boron Nitride Nanosheets for High‐Performance Aramid‐Based Dielectric Films with Enhanced Multifunctionality

**DOI:** 10.1002/advs.202516944

**Published:** 2025-10-24

**Authors:** Zi Wang, Chao Bian, Jia‐Cheng Zhang, Meng Gao, Ying‐Ying Tong, Jun‐Xue Chen, Lin Zhang, Ran Zhuo, Jun‐Wen Ren, Jun‐Wei Zha, Shen‐Li Jia

**Affiliations:** ^1^ Clean Energy Power Systems and Equipment Key Laboratory of Sichuan Province, College of Electrical Engineering Sichuan University Chengdu 610065 P. R. China; ^2^ China Southern Power Grid CSG Electric Power Research Institute Guangzhou 610213 P. R. China; ^3^ State Key Laboratory of Alternate Electrical Power System with Renewable Energy Sources, School of Electrical and Electronic Engineering North China Electric Power University Beijing 102206 P. R. China

**Keywords:** aramid nanofibers, boron nitride nanosheets, interfacial bonding, plasma treatment, power electronic devices, thermal conductivity

## Abstract

Aramid paper, due to its lightweight structure, mechanical strength, and excellent dielectric performance, is widely employed in insulation systems of electronic devices and high‐voltage equipment. However, its inherently poor thermal conductivity (*λ*) restricts its applicability in modern high‐power systems with demanding thermal management needs. Additionally, conventional blending approaches often yield poor filler‐matrix interfaces, which severely limit the enhancement of *λ* and simultaneously deteriorate other properties. Herein, a plasma‐assisted amino functionalization approach is reported for boron nitride nanosheets to reinforce its interfacial affinity with 1D aramid nanofibers (ANF). Together with Silk Fibroin (SK), serving as a flexible molecular binder, a biomimetic nacre‐inspired architecture is achieved through a self‐assembly process. The synergistic effect of strong interfacial interactions and a 3D hydrogen bonding network endows the composite films with outstanding thermal conductivity of 13.89 Wm^−1^K^−1^, excellent tensile strength of 307.08 MPa, as well as superior thermal resistance and long‐term operational stability. Moreover, the highly ordered microstructure results in an ultrahigh breakdown strength (up to 430 kVmm^−1^) and a low dielectric loss. The findings of this study provide a rational design strategy for multifunctional polymer‐based dielectric materials aimed at next‐generation high‐power electronic devices.

## Graphical abstract

A bio‐inspired strategy is proposed to fabricate high‐performance composite films by plasma‐assisted amino‐functionalized boron nitride nanosheets (BNNS‐NH_2_) with aramid nanofibers (ANF) and Silk Fibroin (SK). The synergistic effect of a 3D hydrogen‐bonding network and strong interfacial adhesion results in superior thermal conductivity of 13.89 Wm^−1^K^−1^, tensile strength of 307.08 MPa, breakdown strength (up to 430 kV mm^−1^), excellent thermal reliability and dielectric stability.

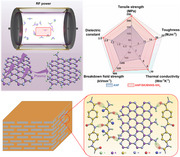

## 1 Introduction

Owing to its light weight, ease of processing, and outstanding thermal stability and flexibility, aramid paper has become key insulating materials in modern power electronic devices, such as electric vehicle motors, high overload transformers, and wind power generators.^[^ ^1^, ^2^, ^3^, ^4^ ^]^ However, the pursuit of higher efficiency, compact integration, and elevated power density in modern electronic devices has posed critical challenges to conventional insulating materials.^[^ ^5^ ^]^ For example, to satisfy the demands of new energy vehicles for high power output, fast charging, and operational safety and reliability, motor insulation materials must maintain high performance under the coupled and superimposed stresses such as high temperature, strong electric fields, and high‐frequency pulses.^[^ ^6^, ^7^, ^8^, ^9^ ^]^ Despite its favorable dielectric and mechanical properties, aramid paper suffered from intrinsically poor thermal conductivity, which significantly limited its heat dissipation performance.^[^ ^10^ ^]^ Inefficient heat dissipation caused excessive temperature rise in insulating materials, accelerating thermal aging and potentially triggering thermal breakdown, which significantly compromised the reliability and longevity of the equipment. Therefore, it is imperative to develop novel dielectric composite paper that combine excellent thermal conductivity, outstanding insulation properties, and high‐temperature resistance to meet the demands of rapidly advancing compact, high‐power power electronic devices.

To enhance thermal conductivity, highly thermally conductive nanofillers were incorporated into the polymers,^[^ ^11^, ^12^ ^]^ such as ceramic fillers,^[^ ^13^ ^]^ metal fillers,^[^ ^14^ ^]^ carbon‐based fillers,^[^ ^12^, ^15^, ^16^, ^17^ ^]^ etc. In particular, boron nitride nanosheets (BNNS), as a representative ceramic material, possess ultrahigh thermal conductivity (≈2000 Wm^−1^K^−1^), wide bandgap (≈5.9 eV), and high dielectric breakdown strength (≈800 kVmm^−1^), providing an ideal filler for thermally conductive and electrically insulating polymer composites.^[^ ^18^, ^19^ ^]^ However, numerous studies have shown that the enhancement in thermal conductivity is significantly lower than the theoretical predictions by incorporating BNNS.^[^ ^20^ ^]^ This is primarily attributed to the smooth surface and chemically inert of BNNS, which hinders the formation of strong interfacial interactions with the polymer matrix.^[^ ^21^ ^]^ The enormous number of micro/nano interfaces within composite materials leads to intense phonon scattering, resulting in high interfacial thermal resistance.^[^ ^22^ ^]^ In addition, filler agglomeration resulting from surface inertness also adversely impacts the processability of the material. Surface modification is an effective strategy to enhance the surface activity of BNNS.^[^ ^23^, ^24^ ^]^ Based on the type and strength of intermolecular chemical bonding, surface modification approaches can be classified into covalent grafting and non‐covalent modification. Non‐covalent modification promotes the interface interaction between the matrix and filler by introducing weak interactions such as electrostatic forces, van der Waals forces, and π‐π stacking.^[^ ^25^ ^]^ Liu et al. achieved non‐covalent modification of boron nitride using polyetherimide by leveraging the interactions between nitrogen atoms on the polyetherimide chains and the hexagonal boron nitride (h‐BN) surface, as well as the adsorption between electrons and vacant orbitals.^[^ ^26^ ^]^ This modification improved the interfacial compatibility between h‐BN and Polyetheretherketone, and reduced phonon scattering caused by interfacial voids. Unfortunately, non‐covalent modification layers generally suffer from poor stability, as the weak interactions involved tend to degrade or fail under harsh conditions such as high temperatures. In contrast, covalent grafting offers significantly greater stability (permanent vs reversible) and stronger interfacial bonding (covalent bonds vs weak interactions).^[^ ^27^ ^]^ However, even the more efficient covalent grafting method still exhibits diminishing returns.^[^ ^28^ ^]^ In particular, excessive functionalization may introduce defects and phonon scattering centers into BNNS, thereby impairing their intrinsic characteristics. Moreover, covalent grafting typically requires relatively complex processing steps.

Recently, plasma surface treatment has been widely recognized as an efficient covalent grafting strategy because of its operational simplicity and environmental friendliness.^[^ ^29^, ^30^ ^]^ Upon plasma discharge, nitrogen‐containing gases such as NH_3_, NH_3_/Ar, N_2_, and N_2_/H_2_ release highly reactive species (e.g., N, NH_x_, H) that can interact with material surfaces, enabling mild etching while grafting functional group.^[^ ^31^, ^32^, ^33^ ^]^ Notably, With carefully optimized plasma parameters such as discharge power and treatment duration, surface modification can effectively introduce functional groups while maintaining the intrinsic crystallinity and planar structure of the substrate. For example, Di‐Oliveira et al. reported that oxygen plasma treatment introduced ‐OH and ‐COOH groups onto graphene, markedly improving surface wettability without disrupting the sp^2^‐hybridized structure.^[^ ^34^ ^]^ Yang et al. performed oxygen plasma pretreatment before grafting synthetic ammonia functional groups on carbon fibers and increased the oxygen plasma treatment time from 0 to 3 min, which significantly improved the interfacial bonding and mechanical properties of the composites.^[^ ^35^ ^]^ Clearly, plasma treatment is an effective approach for covalent modification, enabling stronger filler‐matrix interfacial interactions through functional group introduction and markedly improving the thermal conductivity of composites.

In addition to high thermal conductivity, insulation performance, thermal stability, and mechanical properties are also critical parameters for evaluating dielectric materials in power devices.^[^ ^36^, ^37^, ^38^ ^]^ However, these properties are governed by different physical mechanisms, and in particular, thermal conductivity and electrical insulation often exhibit a trade‐off relationship. Bioinspired micro‐structural design offers a promising approach to overcoming this contradiction.^[^ ^39^, ^40^, ^41^, ^42^ ^]^ For example, inspired by the micro‐structure of nacre, Yu et al. fabricated a highly ordered “brick‐and‐mortar” structure by compressing a mixture of TiO_2_‐coated mica nanosheets (TiO_2_‐mica) and nanocellulose. This bioinspired architecture enabled the organic integration of high strength (281 MPa), high toughness (11.5 MPa·m^1/2^), high stiffness (20 GPa), and excellent thermal resistance.^[^ ^43^ ^]^ It is evident that constructing well‐organized and rational microstructures is an effective approach to enhancing the overall performance of composite materials. 1D nanofibers are widely used as fundamental building blocks in bioinspired microstructures due to their extremely high aspect ratio, large specific surface area, and pronounced nanoscale effects.^[^ ^44^, ^45^ ^]^ Among them, aramid nanofibers (ANF) not only inherit the excellent mechanical strength and high thermal stability of aramid fibers, but also possess a greater abundance of surface‐active functional groups, making them one of the most promising matrix materials for advanced dielectric composites.^[^ ^46^, ^47^, ^48^, ^49^ ^]^

In this study, 1D ANF and 2D amino‐functionalized boron nitride nanosheets (BNNS‐NH_2_) were combined to construct high‐performance nacre‐inspired composite films via vacuum‐assisted filtration and hot‐pressing self‐assembly. Plasma surface treatment enhanced the surface activity of BNNS while preserving its intact crystalline structure, thereby avoiding the bottleneck effect caused by excessive functionalization. In addition, flexible Silk Fibroin (SK) molecules were introduced to intertwine with rigid ANF and BNNS‐NH_2_, forming a 3D hydrogen bonding network that significantly enhanced their interfacial interactions. Within the ANF/SK framework, the ordered alignment of BNNS‐NH_2_ effectively improved phonon transport pathways, charge dissipation, and stress transfer. As a result, the composite films exhibited excellent mechanical properties, high thermal conductivity, and outstanding dielectric performance. The results indicated that the fabricated bioinspired films have great potential to meet the stringent material requirements of modern electronic and power devices, and provided both theoretical and experimental foundations for the development of high‐performance polymer‐based dielectric materials.

## 2 Results and Discussion

**Figure 1:**
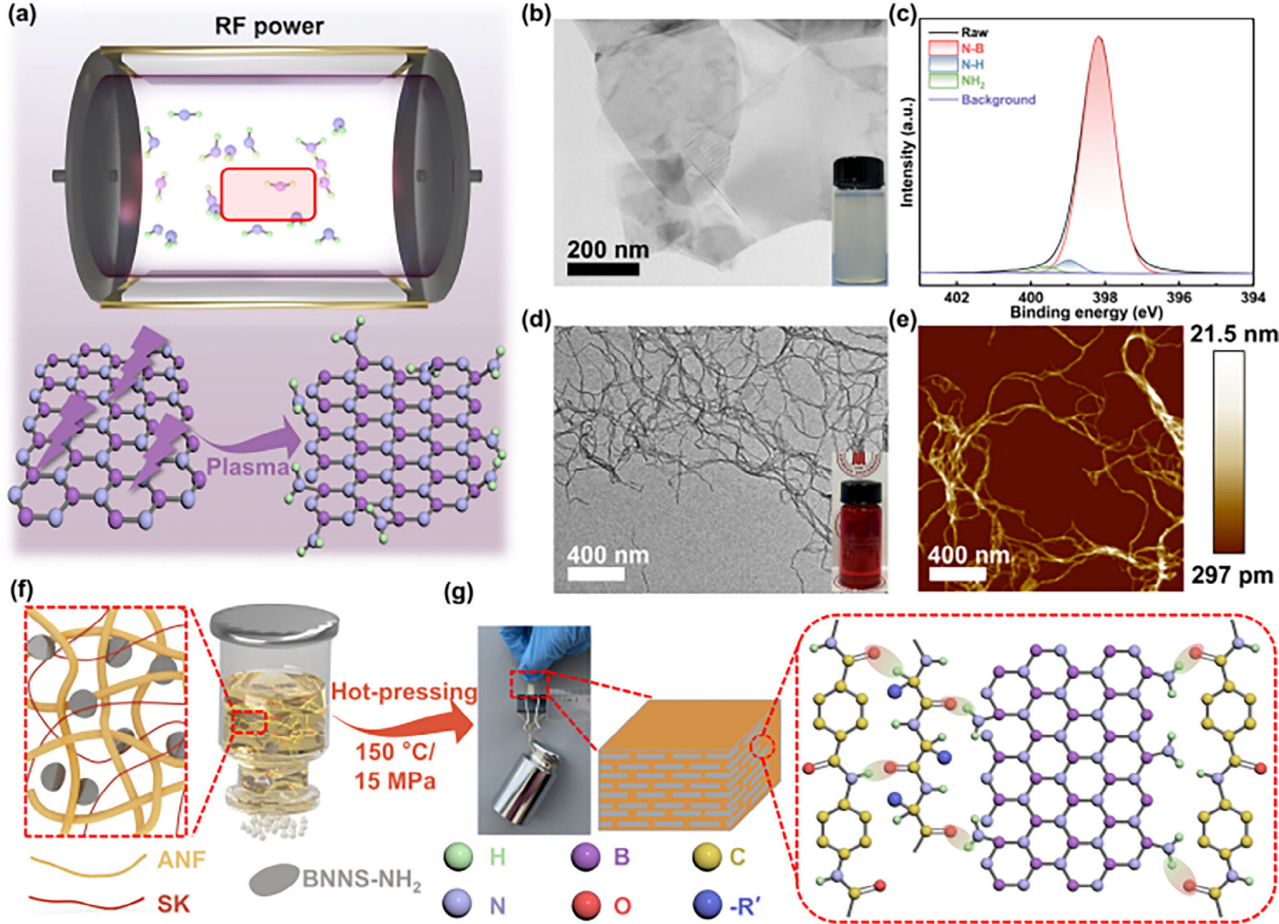
a) Illustration of the process of grafting amino groups on the surface of BNNS. b) TEM image of BNNS (inset:digital image of BNNS suspensions.). c) XPS analysis of BNNS‐NH_2_. d) TEM image of ANF (inset: digital image of ANF suspensions.). e) AFM image of ANF. f) Fabrication of ANF/SK/BNNS‐NH_2_ composite films via vacuum‐assisted filtration. g) Schematic showing the microstructure of ANF/SK/BNNS‐NH_2_ composite films.

BNNS‐NH_2_ was fabricated by ion intercalation‐assisted exfoliation followed by plasma surface treatment, as shown in **Figure** **1**a. First, in comparison with the multilayer‐stacked h‐BN (Figure S3a, Supporting Information), BNNS (Figure 1b; Figure S3c, Supporting Information) exhibited an ultrathin, transparent, and few‐layered structure. Wrinkling and edge folding, which are commonly associated with reduced nanosheet thickness, were also observed in the low‐resolution SEM images (Figure S3b, Supporting Information). According to AFM measurements (Figures S3d,e, Supporting Information), the thickness of BNNS was ≈1.658 nm, directly evidenced the structural modification. XRD analysis (Figure S3f, Supporting Information) showed a noticeable shift in the diffraction peak of BNNS from 26.72 to 26.68°, indicating an increased interlayer spacing and reduced thickness.^[^ ^50^ ^]^ In addition, compared to h‐BN, the in‐plane high‐frequency vibrational mode (E2g) of BNNS (Figure S3g, Supporting Information) demonstrated a significant decrease in intensity, confirming the weakened interlayer interactions due to exfoliation.^[^ ^51^ ^]^ All these experimental results collectively confirmed the successful preparation of BNNS. After BNNS stripping was prepared, the BNNS surface was treated with plasma for amino functionalization. The highly active plasma ionized or excited the ammonia and formed particle products (NH_3_, NH, N, H, etc.). These particle products carried high energy during the plasma treatment and hit the BNNS surface, causing cross‐linking or etching and breaking of the edge B─N bonds. The broken molecular chain then interacted with free H and N atoms in the plasma and introduced new ‐NH_2_ groups(Figure 1a). The surface amination of BNNS was confirmed by XPS analysis (Figure 1c), where the BNNS‐NH_2_ sample exhibited an N1s peak at 397.9 eV, attributed to N─B, N─H, and ‐NH_2_ groups.^[^ ^52^ ^]^ FTIR spectroscopy further supported this observation, showing an additional absorption peak around ≈3433 cm^−1^ in BNNS‐NH_2_ (Figure S4a, Supporting Information), corresponding to the N─H stretching vibration.^[^ ^53^ ^]^ In addition, TGA curves revealed that while pristine BNNS exhibited a minimal weight loss of only 0.2% at 800 °C, BNNS‐NH_2_ showed a weight loss of 1.3% (Figure S4b, Supporting Information), which was attributed to the thermal decomposition of grafted amino groups on the BNNS surface. These results collectively confirmed the successful grafting of ‐NH_2_ groups. The resulting BNNS‐NH_2_ also demonstrated excellent dispersibility in aqueous solution and exhibited a strong Tyndall effect (Figure S5, Supporting Information). The structural features of BNNS‐NH_2_ were investigated (Figure S4c,d, Supporting Information), and it was found that the nanosheet dimensions were largely preserved following plasma treatment. In addition, XRD analysis (Figure S4e,f, Supporting Information) showed that BNNS‐NH_2_ retains a pronounced (002) peak, with only slight changes in peak intensity and full width at half maximum compared with BNNS. The results demonstrated that plasma‐assisted amination did not disrupt the intact crystal structure of BNNS, while the introduction of ‐NH_2_ groups effectively enhanced the interfacial interactions between BNNS‐NH_2_ and the matrix. The preparation of ANF followed the method proposed by Yang et al (Figure S6a, Supporting Information).^[^ ^54^ ^]^ The intermolecular hydrogen bonding interactions (via amide groups) between aramid fiber molecular backbones were weakened due to the deprotonation effect.^[^ ^55^ ^]^ Under electrostatic repulsion, the hydrogen bonds between molecular chains gradually break, leading to complete dissociation and dispersion. The aramid fibers exhibited a very smooth surface with a diameter of ≈18 µm (Figure S6b, Supporting Information). The resulting ANF exhibited lengths of several micrometers (Figure 1d) and diameters of ≈20 nm (Figure 1e), featuring a high aspect ratio and stable dispersion in DMSO/KOH (in the inset of Figure 1e). Subsequently, the ANF/DMSO dispersion was thoroughly mixed with the BNNS‐NH_2_/DMSO dispersion, followed by washing with deionized water to remove DMSO and KOH. The well‐ordered composite films were then fabricated via vacuum filtration‐induced self‐assembly (Figure 1f,g).

**Figure 2:**
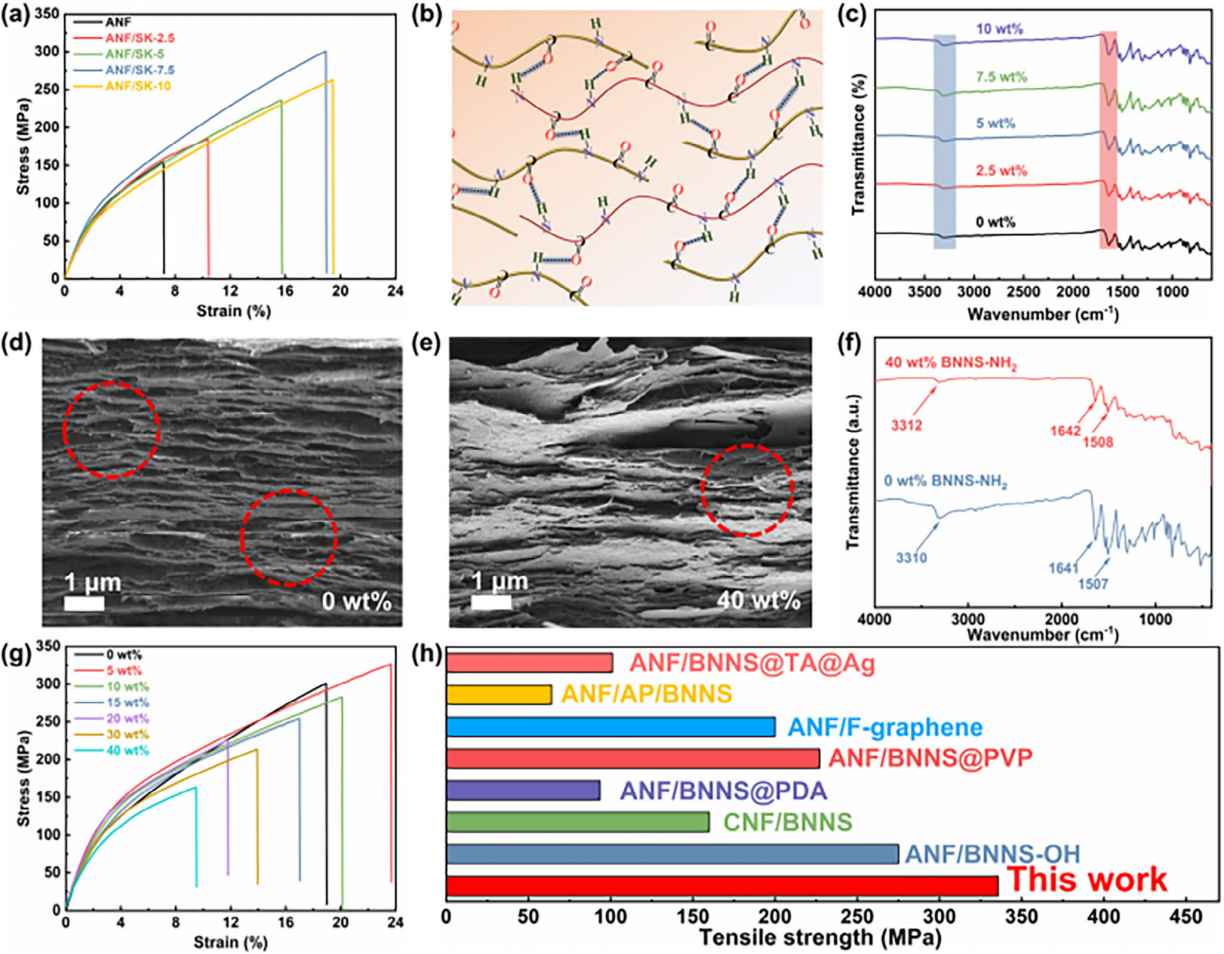
Mechanical properties and microstructure of ANF/SK and ANF/SK/BNNS‐NH_2_ composite films. a) Stress‐strain curves of ANF/SK films with different SK concentrations. b) Schematic of hydrogen bonding interactions between ANFs and SK. c) FT‐IR spectra of ANF/SK films with different SK concentrations. d) Stress‐strain curves of composite films with different BNNS‐NH_2_ concentrations. SEM images of cross‐sections of e) ANF‐only and f) 40 wt.% BNNS‐NH_2_ composite films of ANF/SK/BNNS‐NH_2_. g) FT‐IR spectra of composite films with 0 and 40 wt.% BNNS‐NH_2_ concentrations. h) Comparison of the tensile strength of ANF/SK/BNNS‐NH_2_ composite membranes with those that have been reported.

Herein, we introduced flexible SK into the rigid ANF matrix through interfacial optimization design, forming a “rigid‐flexible” polymer bimolecular network to enhance the mechanical performance of the composite films. As shown in **Figure** **2**a, the incorporation of SK significantly improved the mechanical properties of the composite films. Notably, at a SK concentration of 7.5 wt.%, the tensile strength, elongation at break, and toughness of the composite film increased markedly compared to the pure ANF film (307.08 MPa vs 163.98 MPa, 18.55% vs 10.06%, and 38.00 MJ m^−3^ vs 11.20 MJ m^−3^), representing enhancements of 87.27%, 84.39%, and 239.29%, respectively (Figure S7a,b, Supporting Information). This significant improvement was primarily attributed to the formation of a large number of intermolecular hydrogen bonds between the amide groups of ANF and the peptide bonds of SK (Figure 2b), which absorbed energy during fracture and thereby enhanced the mechanical strength of the composite films. This mechanism was further confirmed by FT‐IR spectroscopy. As shown in Figure 2c, the N─H and C═O stretching vibration peaks of the ANF/SK film shifted to higher wavenumbers compared to those of the pure ANF film (3300 and 1640 cm^−1^, respectively). Specifically, at the SK content of 7.5 wt.%, the peak shifts were most pronounced, reaching 3310 and 1641.16 cm^−1^, respectively (Figure S7c, Supporting Information), indicating the strongest hydrogen bonding. Consistent with the mechanical testing results, the composite films exhibited the best mechanical performance at a SK concentration of 7.5 wt.%, and this configuration was therefore adopted for the subsequent preparation of ANF/SK/BNNS‐NH_2_ ternary composite films.

As shown in Figure S8 (Supporting Information), the fabricated ANF/SK/BNNS‐NH_2_ composite film could be easily bent, demonstrating excellent flexibility. AFM imaging revealed a smooth film surface without noticeable defects (Figure S9a, Supporting Information). Figure 2d,e presented cross‐sectional SEM images of the ANF/SK film and the ANF/SK/BNNS‐NH_2_ composite film with 40 wt.% BNNS‐NH_2_ concentration. The composite films exhibited a characteristic “brick‐and‐mortar” architecture, where the ANF/SK network served as the “mortar” and the BNNS‐NH_2_ acted as “bricks”, being intertwined and stacked in layers. As shown in Figure 2d, prominent fiber‐bridging structures were observed between the dense layers. These bridges helped dissipate more energy during tearing damage, thereby providing strong reinforcement for the film's high tensile strength. In addition, BNNS‐NH_2_ formed hydrogen bonds with both ANF and SK (Figure 1h), contributing to a denser hydrogen bonding network. FT‐IR spectra (Figure 2f) indicated that the film containing BNNS‐NH_2_ exhibited a stronger absorption peak around ≈3312 cm^−1^ corresponding to N─H stretching. Furthermore, the characteristic peaks corresponding to the amide I (≈1642 cm^−1^) and amide II (≈1508 cm^−1^) bands showed a noticeable blue shift compared to the film without BNNS‐NH_2_, indicating enhanced hydrogen bonding interactions. The mechanical property trends of composite films with different BNNS‐NH_2_ contents further supported this analysis. As shown in Figure 2f, the mechanical properties reached their peak at a filler concentration of 5 wt.%, with tensile strength, elongation at break, and toughness reaching 335.72 MPa, 23.92%, and 55.29 MJ m^−3^, respectively (Figure S9b,c, Supporting Information). Subsequently, a decline in mechanical performance was observed, which was attributed to increased filler aggregation at higher BNNS‐NH_2_ loading, leading to stress concentration points within the films.^[^ ^56^ ^]^ Nevertheless, owing to the well‐designed 3D hydrogen bonding network, the ANF/SK/BNNS‐NH_2_ composite films still maintained a tensile strength of 166 MPa even at 40 wt.% BNNS‐NH_2_ loading. At the optimal filler concentrations of 5 and 10 wt.%, the mechanical properties of the composite films before and after plasma treatment were compared (Figure S9d, Supporting Information). Although the incorporation of SK constructed a rich hydrogen‐bonding network within the composite, a modulus mismatch still existed between the rigid BNNS and the polymer matrix. The introduction of ‐NH_2_ groups enabled the formation of hydrogen bonds at the filler–matrix interface, increasing the energy required for film fracture. As a result, ANF/SK/BNNS‐NH_2_ composite films exhibited significantly improved mechanical properties compared with ANF/SK/BNNS films at the same filler concentrations, confirming the effectiveness of the plasma surface treatment. Compared with previously reported ANF‐based composite films, the films developed in this work also exhibited superior mechanical performance (Figure 2h; Table S1, Supporting Information).^[^ ^57^, ^58^, ^59^, ^60^, ^61^, ^62^, ^63^ ^]^

**Figure 3:**
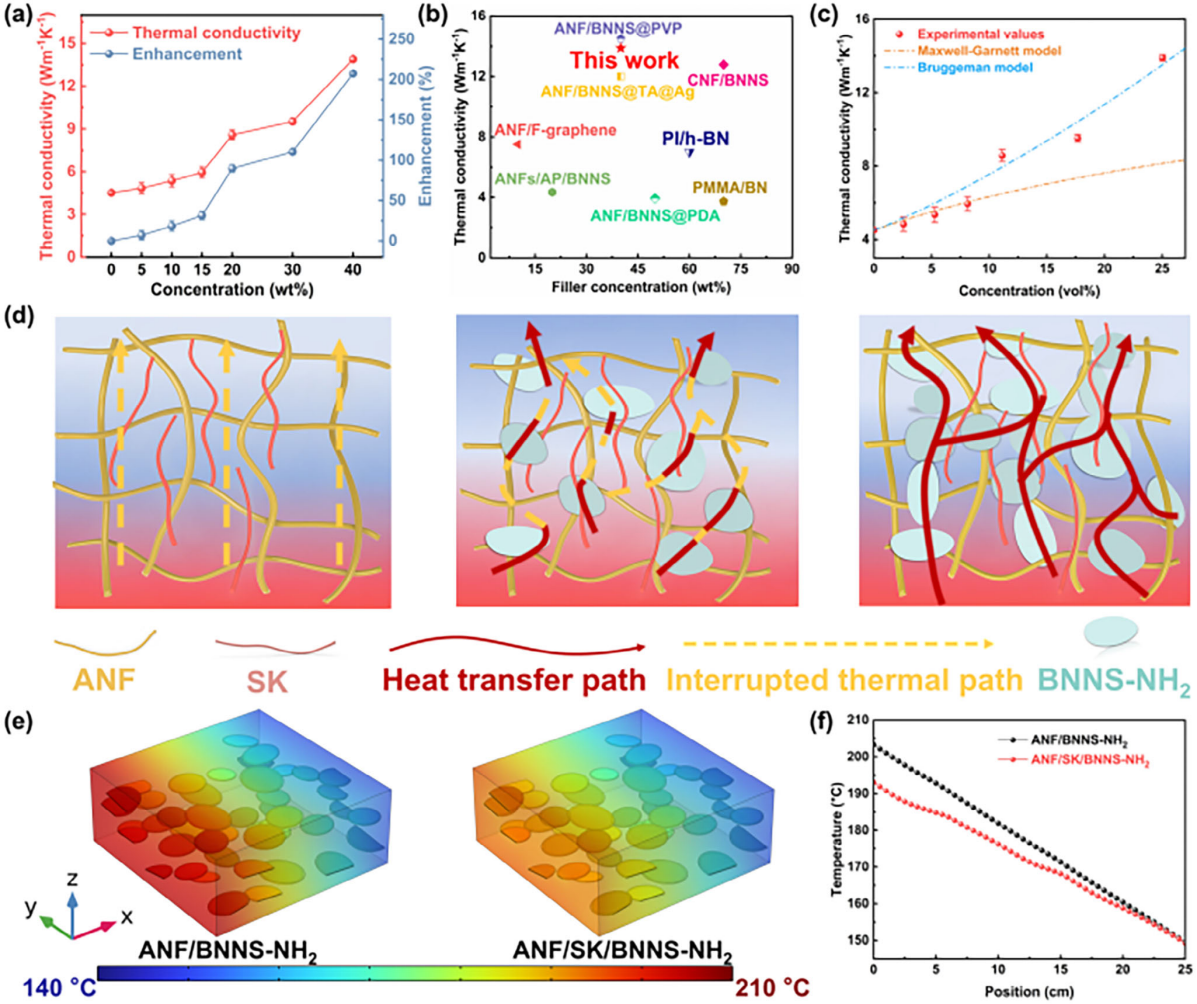
Thermally conductive properties of ANF/SK/BNNS‐NH_2_ composite films. a) The *λ* of composite films with different BNNS‐NH_2_ concentrations. b) Comparison of this work with other work reported. c) The experimental *λ* of composite films and theoretical values simulated by the MG‐EMT model and Burggrman model. d) Heat transfer path of ANF/SK/BNNS‐NH_2_ composite films. e) Steady‐state temperature distribution of ANF/BNNS and ANF/SK/BNNS‐NH_2_ composite films. f) Corresponding temperature variation along the direction of heat transfer.

**Figure** **3**a illustrated the thermal conductivity values (*λ*) of ANF/SK/BNNS‐NH_2_ composite films. To further evaluate the λ enhancement efficiency of BNNS‐NH_2_ on the composite films, the growth rate of thermal conductivity (Thermal Conductivity Enhancement, TCE) of the composite films was defined as follows^[^ ^64^ ^]^:

(1) $$\mathrm{TCE}=\left(\lambda_{c}-\lambda_{m}\right)/\lambda_{m}$$

where *λ*_c_ and *λ*_m_ are the thermal conductivities of ANF/SK/BNNS‐NH_2_ and ANF/SK composite films, respectively. The *λ* of composite films significantly increased with concentration of BNNS‐NH_2_. In particular, the ANF/SK/BNNS‐NH_2_ films with 40 wt.% content achieved a high *λ* of 13.89 Wm^−1^K^−1^, which was 207.30 % higher than that of ANF/SK film (4.52 Wm^−1^K^−1^). Compared with other paper‐based materials reported in the literature, the ANF/SK/BNNS‐NH_2_ composite film exhibited a significant competitive advantage in terms of *λ* (Figure 3b).^[^ ^52^, ^57^, ^58^, ^60^, ^61^, ^62^, ^63^, ^65^ ^]^ The interfacial thermal resistance (*R*_k_) of the composite films was further investigated using a modified Maxwell‐Garnett effective medium theory (MG‐EMT) model and Bruggeman model (the formula is provided in Section S10 of the Supporting Information). In the low filler content region (0–15 wt.%), the calculated *R*_k_ of different composite films was fitted using the modified MG‐EMT model, which accounts for the geometry and distribution of 2D fillers. As shown in Figure S10c (Supporting Information), after plasma treatment, the *R*_k_ of ANF/SK/BNNS‐NH_2_ films decreased from 6.541 × 10^−8^ to 1.896 × 10^−8^ m^2^WK^−1^. The introduction of amino functionalization effectively enhanced the interfacial bonding between BNNS and the ANF/SK matrix, thereby suppressing interfacial phonon scattering. This explains why, at the same filler loading, the ANF/SK/BNNS‐NH_2_ films exhibited higher *λ* than the ANF/SK/BNNS films (Figure S10b, Supporting Information). In the high filler content region (20–40 wt.%), the Bruggeman model was applied to calculate interfacial thermal resistance. As shown in Figure 3c, with increasing filler content, the calculated *R*_k_ further decreased from 1.896 × 10^−8^ to 4.246 × 10^−9^ m^2^WK^−1^. Figure 3d illustrated the mechanism by which the incorporation of BNNS‐NH_2_ enhanced the *λ* of the composite films. The excellent *λ* of the ANF/SK/BNNS‐NH_2_ composite films was attributed to two main factors. First, due to the “brick‐and‐mortar” structure, the high‐aspect‐ratio BNNS‐NH_2_ were horizontally aligned within the film, which increased the contact probability between fillers and facilitated the construction of continuous and efficient phonon transport pathways. Second, the strong hydrogen bonding between BNNS‐NH_2_ and the ANF/SK matrix effectively reduced the interfacial thermal resistance. To further validate the contribution of interfacial interactions to the efficiency of *λ* enhancement, finite element simulations were performed to model the steady‐state temperature rise of two composite films with identical filler concentration (Figure S11a,b, Supporting Information). The steady‐state temperature distributions of the ANF/BNNS‐NH_2_ film and the ANF/SK/BNNS‐NH_2_ film under the equal heat flux conditions were shown in Figure 3e. Owing to improved interfacial bonding, phonon scattering was effectively suppressed, and the heat flux density at the interface was significantly enhanced (Figure S11c,d, Supporting Information). Consequently, the ANF/SK/BNNS‐NH_2_ composite film exhibited a prominently lower steady‐state temperature along the heat flow direction compared to the ANF/BNNS‐NH_2_ film with weak interfacial interactions (Figure 3f), demonstrating superior thermal conductivity.

**Figure 4:**
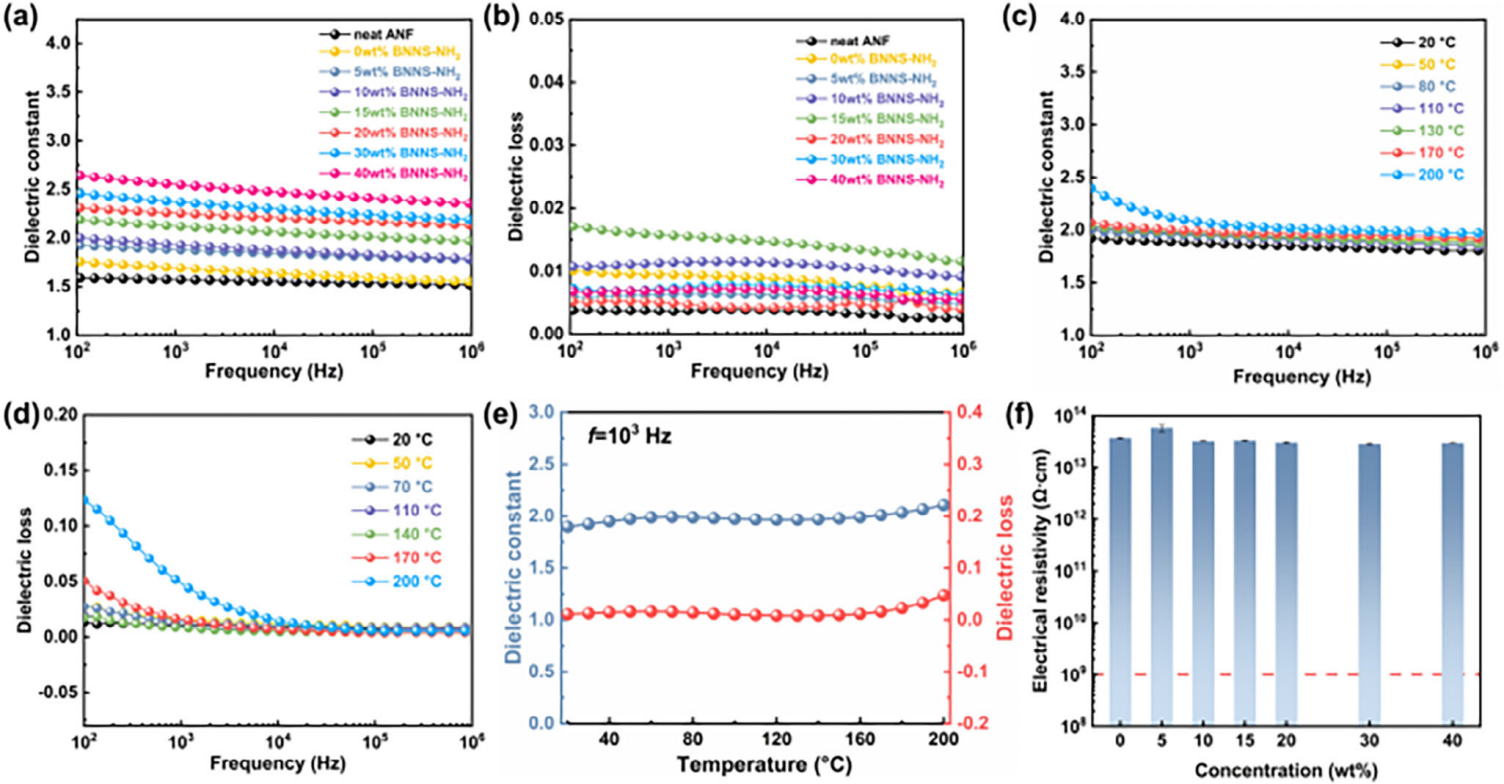
Dielectric properties of ANF/SK/BNNS‐NH_2_ composite films. a) Dielectric constant, b) dielectric loss tangent of composite films with different BNNS‐NH_2_ concentrations. c) Dielectric constant, d) dielectric loss tangent of ANF/SK/BNNS‐NH_2_ composite films containing 15 wt.% BNNS‐NH_2_ at different temperatures. e) Variation of dielectric properties of ANF/SK/BNNS‐NH_2_ composite films containing 15 wt.% BNNS‐NH_2_ with temperature at 10^3^ Hz. f) Volume resistivity of pure ANF and ANF/SK/BNNS‐NH_2_ composite films.

**Figure** **4** shows the dielectric properties of the composite films with varying BNNS‐NH_2_ concentration. Within the frequency range of 10^2^ to 10^6^ Hz, the dielectric constant of the ANF/SK/BNNS‐NH_2_ composite films exhibited an increasing trend with the rising filler content. This increase was primarily attributed to interfacial polarization caused by the mismatch in dielectric constants between BNNS‐NH_2_ and ANF, which led to charge carrier accumulation near the interfaces (Figure 4a). Moreover, the intermolecular hydrogen bonding among ANF, SK, and BNNS‐NH_2_ facilitated tight entanglement, resulting in a denser film structure and a slight further increase in dielectric constant.^[^ ^66^ ^]^ When the filler concentration reached 40 wt.%, the dielectric constant of the composite film increased only to 2.64 while still maintaining excellent frequency stability. In addition, the dielectric loss and AC conductivity of the composite films remained extremely low over a broad frequency range (Figure 4b; Figure S12a, Supporting Information).Temperature was recognized as a critical factor influencing the service performance of polymer‐based insulating materials.^[^ ^67^ ^]^ The ANF/SK/BNNS‐NH_2_ composite film with the highest tensile strength (5 wt.% BNNS‐NH_2_) was selected to investigate the effect of temperature on its dielectric properties. Figure 4c,d and S12b (Supporting Information) presented the frequency‐dependent dielectric behavior of the composite films at various temperatures. The dielectric constant showed a slight increase with rising temperature. Both the dielectric loss and AC conductivity remained nearly unchanged within the temperature range from room temperature to 140 °C. However, when the temperature increased to 170 °C and above, the dielectric loss and AC conductivity exhibited a significant rise at low frequencies (10^2^–10^3^ Hz), while remaining nearly constant at high frequencies. This phenomenon was attributed to the lag of interfacial polarization relative to the variation in the external electric field. Figure 4e illustrated the temperature dependence of dielectric properties at 10^3^ Hz. At 200 °C, the composite film maintained low values of dielectric constant and loss tangent (2.1 and 0.05, respectively), indicating excellent thermal stability. The volume resistivity of the composite films is shown in Figure 4f. Variations in BNNS‐NH_2_ concentration had minimal impact on the volume resistivity of the composite films, with all values remaining on the order of 10^13^  Ω·cm‐significantly higher than the defined threshold for insulating materials (10^9^ Ω·cm).

Dielectric breakdown strength was considered a critical parameter for evaluating the performance of polymer‐based insulating materials. The electrical breakdown data of the ANF/SK/BNNS‐NH_2_ composite films were analyzed using a two‐parameter Weibull distribution function, which was expressed by the following equation:

(2) $$P\left(E\right)=1-\exp\left[-{\left(E_{b}/E_{0}\right)}^{\beta }\right]$$

where *P*(*E*) is the cumulative probability of electrical failure, *E*_b_ is the experimentally recorded breakdown field strength, *E*_0_ is the characteristic breakdown field strength at a failure probability of 63.2%, and *β* is the shape parameter of the Weibull modulus distribution, which responds to the dispersion of the breakdown voltage. Each fitted line is linearly described as ^[^ ^59^ ^]^:

(3) $$\ln\left(-\ln\left(1-P\left(E\right)\right)\right)=\beta \ln\left(E_{b}\right)-\beta \left(E_{0}\right)$$

**Figure 5:**
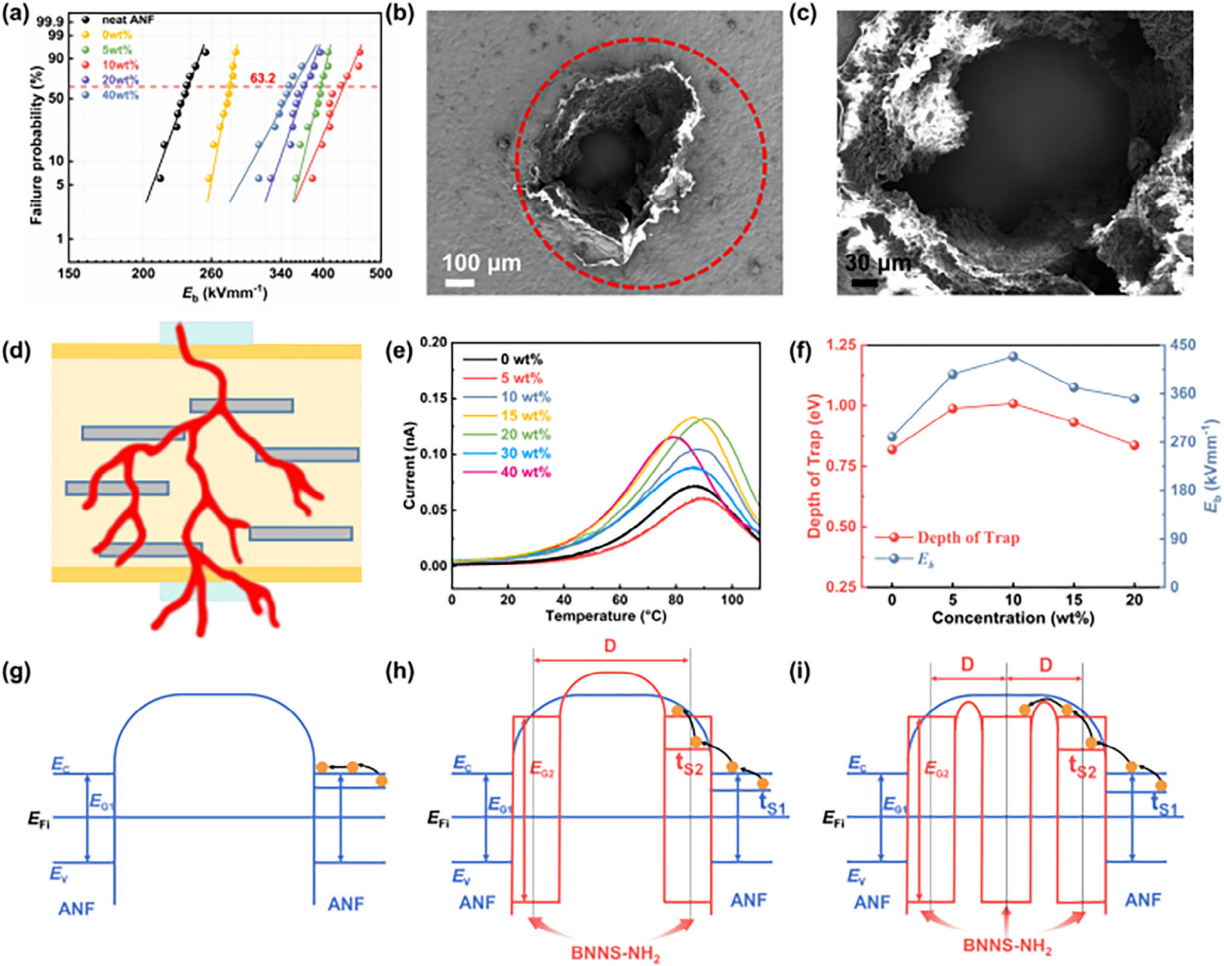
Insulation performance of ANF/SK/BNNS‐NH_2_ composite films. a) Failure probability of dielectric breakdown deduced from the Weibull distribution of neat ANF films and ANF/SK/BNNS‐NH_2_ composite films. b) SEM image and c) its local magnification image of fracture hole in ANF/SK/BNNS‐NH_2_ composite film. d) Schematic diagram of the breakdown path of ANF/SK/BNNS‐NH_2_ composite films. e) TSDC curves and f) breakdown field strength versus trap depth of composite films with different BNNS‐NH_2_ concentrations. g) Energy band structure models of pure ANF films. h) Composite films with moderate addition of BNNS‐NH_2_ and i) excess addition of BNNS‐NH_2_.

The breakdown field strength of the ANF/SK film (279.8 kVmm^−1^) was 18.41% higher than that of the pure ANF film (236.3 kVmm^−1^) (**Figure** **5**a). This improvement was attributed to the interlocking of ANF with flexible molecular chains and the formation of a robust intermolecular hydrogen bonding network, which effectively suppressed the generation of internal microvoids and reduced the injection of free charge carriers.^[^ ^68^ ^]^ The incorporation of high‐dielectric‐strength 2D BNNS‐NH_2_ further enhanced the breakdown strength of the composite films. The ordered embedding of BNNS‐NH_2_ effectively homogenized the internal electric field. Notably, ANF/SK/BNNS films exhibited dielectric breakdown strengths comparable to ANF/SK/BNNS‐NH_2_ films, with the slightly lower values attributed to interfacial defects from poor bonding (Figure S13a, Supporting Information). Moreover, the unique “brick‐and‐mortar” nacre‐inspired structure hindered the propagation of breakdown paths by transforming them from a “unidirectional” to a “branched” configuration, thereby further mitigating electric field localization (Figure 5d ). This mechanism was also supported by the delamination features observed in the SEM images of the breakdown‐damaged composite films (Figure 5b,c). Moreover, in contrast to the “unidirectional” breakdown pattern observed in pure ANF films (Figure S13b, Supporting Information), the “multidirectional” breakdown pattern in the composite films (Figure S13c, Supporting Information) resulted in a significantly larger breakdown pore diameter (≈606 µm vs ≈204 µm).

To further analyze the mechanism by which filler content influenced the breakdown field strength of the composite films, the thermally stimulated depolarization current (TSDC) method was employed to investigate the charge transport and trap characteristics of the films. The discharge curves of composite films with different BNNS‐NH_2_ concentrations are shown in Figure 5e. The original curves were satisfactorily deconvoluted using a double Gaussian function, and the fitting results and corresponding correlation coefficients are presented in Figure S14 (Supporting Information). Based on the deconvoluted TSDC curves, the trap depth (*E*) and trap density (*Q*) were calculated using the following equations:

(4) $$Q=\frac{1}{d}\int_{T_{1}}^{T_{2}}I\left(T\right)dT$$

(5) $$E=\frac{2.47T_{m}^{2}k}{\Delta T}$$

where *d* is the rate of temperature increase, *T*_1_ and *T*_2_ are the start and end temperatures during the warming process, *T*_m_ is the temperature corresponding to the peak current, *∆T* is the temperature difference corresponding to the half peak, k is the Boltzmann constant. The calculated trap parameters for Type I and Type II traps were summarized in Table S2 (Supporting Information). Consistent with the electrical breakdown test results, the trap depth of the films initially increased and then decreased with rising BNNS‐NH_2_ content (Figure 5f). In the pure ANF film, the amorphous regions between adjacent aramid nanofiber crystallites formed a wide and high potential barrier, making it difficult for charge carriers to pass through (Figure 5g). As the filler concentration increased, the interfacial interaction area between BNNS‐NH_2_ and the matrix also expanded. The potential barriers formed at the contact interfaces between the nanofillers and ANF were higher than those in the undoped system, which led to an increase in both trap depth and trap density. Charge carriers were more readily captured by traps during migration, thereby restricting their mobility (Figure 5h).^[^ ^69^ ^]^ Furthermore, the average free path of electrons in the conduction band was shortened. Combined with energy losses during trapping and releasing processes, the formation of high‐energy carriers capable of triggering breakdown was significantly suppressed. However, with further increases in filler content, the spacing between adjacent BNNS‐NH_2_ decreased, leading to mutual interference of their potential fields. As a result, both the width and height of the potential barriers decreased, which in turn reduced some of the trap depths and weakened the suppression of carrier transport.^[^ ^70^ ^]^ Charge carriers could even use shallow traps as stepping stones to reach the conduction band via successive hopping or tunneling effects, ultimately reducing the breakdown strength of the films (Figure 5i).

**Figure 6:**
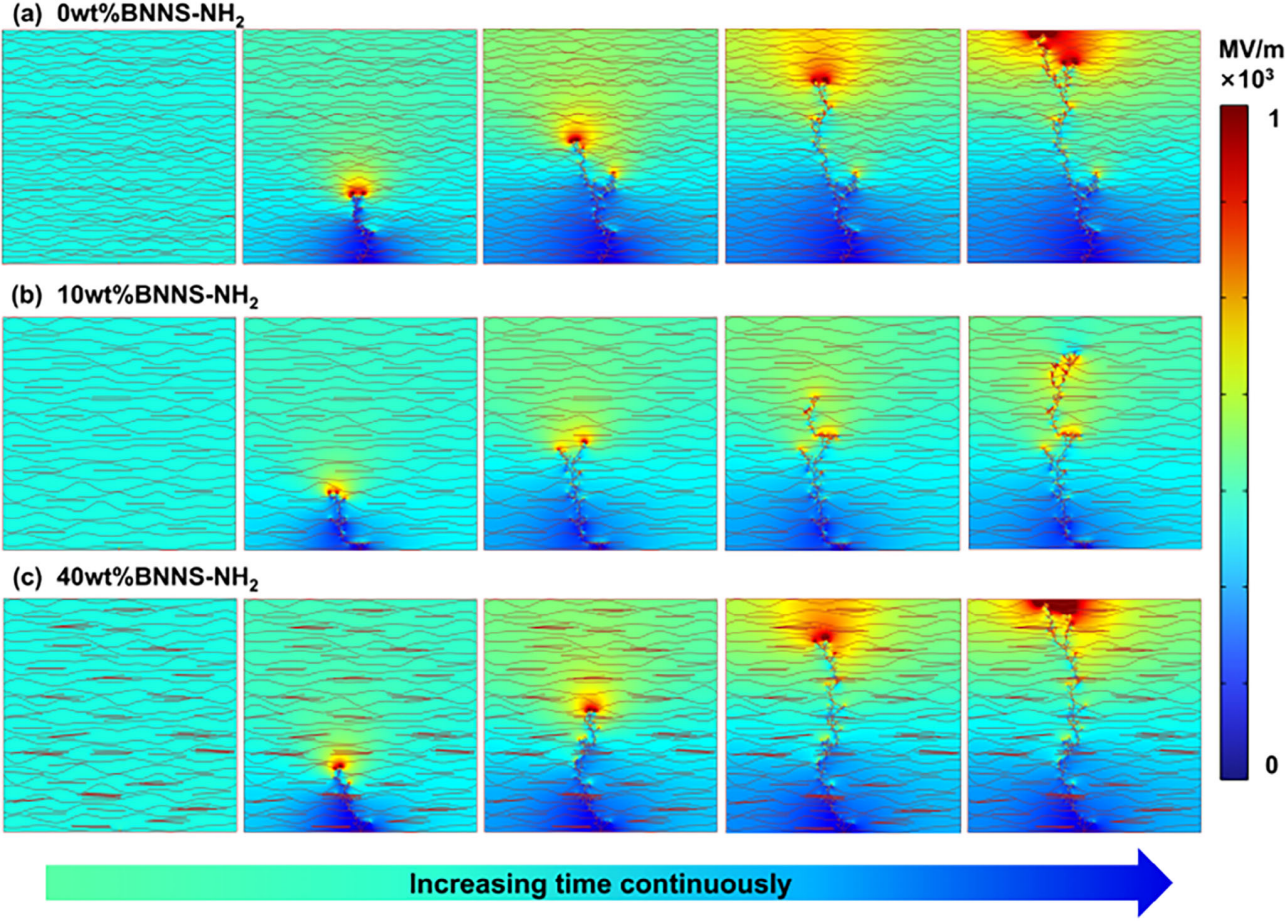
Finite element analysis of electrical breakdown in composite films. a–c) Simulation results of electrical branching development in pure ANF, composite films with different BNNS‐NH_2_ concentrations, respectively.

Finite element simulations (Figure S15, Supporting Information) were conducted to investigate the evolution of electrical breakdown paths in pure ANF films and composite films with the highest breakdown strength and maximum filler concentration (10 and 40 wt.%, respectively). As shown in **Figure** **6**a, the breakdown path in the pure ANF film exhibited a tendency to develop unidirectionally with a relatively rapid propagation rate. In contrast, BNNS‐NH_2_, which possessed high dielectric breakdown strength and was well dispersed within the matrix, forced the breakdown path to circumvent the nanosheets, resulting in a branched development pattern (Figure 6b). The more divergent breakdown paths led to larger breakdown pore diameters, which was consistent with the SEM observations (Figure 5b,c). Moreover, the extended breakdown paths effectively dissipated the energy carried by electrical tree branches, thereby enhancing the dielectric breakdown strength of the films. With a further increase in filler content, the breakdown path morphology transitioned from a “branched” to a “unidirectional” pattern (Figure 6c). Additionally, the inevitable agglomeration caused by excessive filler concentration (Figure S16, Supporting Information) triggered local electric field distortion, resulting in a reduction in dielectric strength. Nevertheless, even at a BNNS‐NH_2_ concentration of 40 wt.%, the characteristic breakdown strength of the composite film remained significantly higher than that of commercially available Nomex insulation paper.

**Figure 7:**
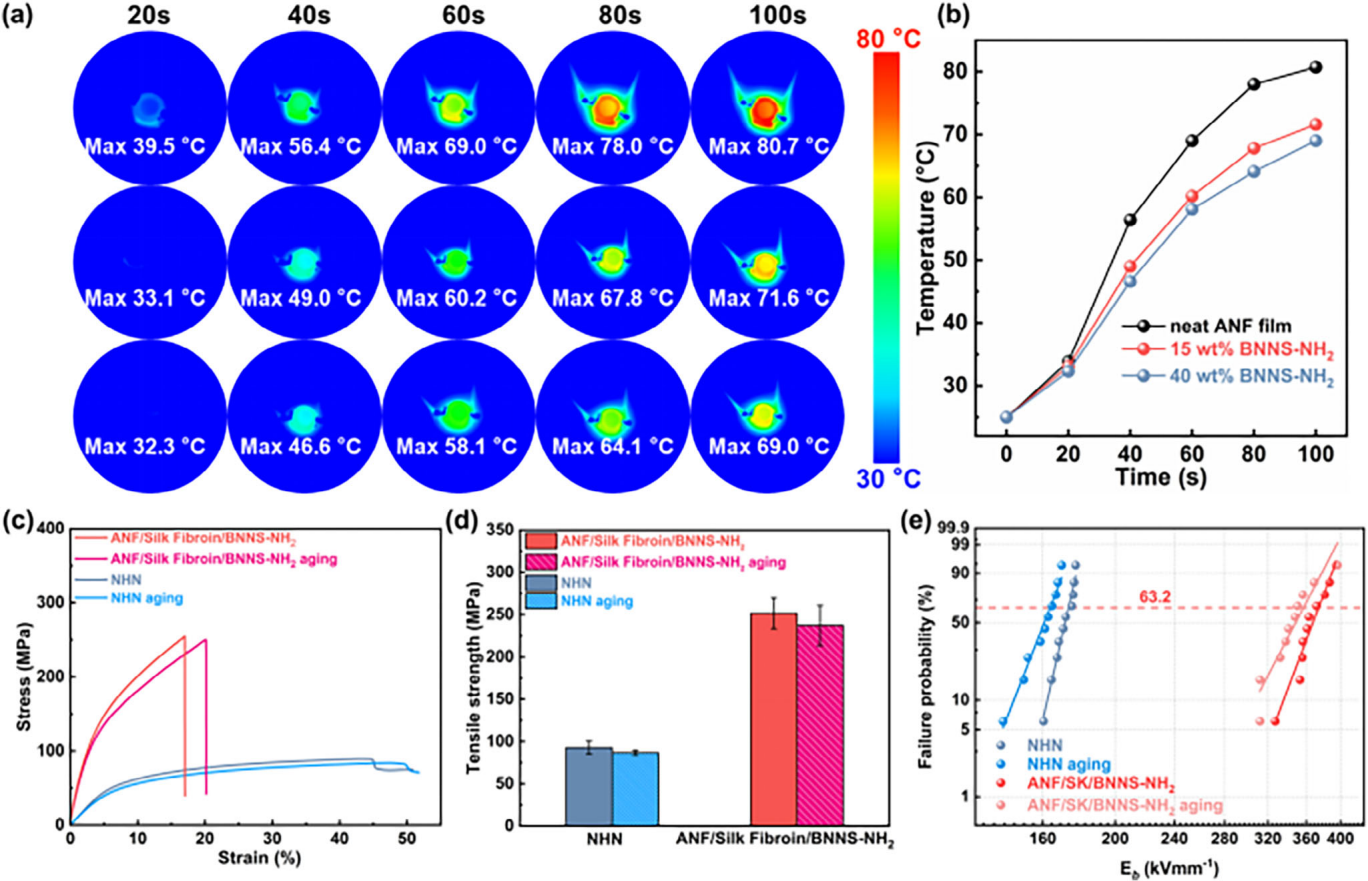
Thermal properties of ANF/SK/BNNS‐NH_2_ composite films. a) Infrared thermal images of a working LED chip with pure ANF film (top), ANF/BNNS‐NH_2_ composite film with 40 wt.% filler loading (middle), and ANF/SK/BNNS‐NH_2_ composite film with 40 wt.% filler loading (bottom) as the thermal substrate. b) Corresponding temperature‐time curves. c) Stress–strain curves of NHN paper, ANF/SK/BNNS‐NH_2_ composite film before and after thermal aging. Comparison of tensile strength d) and characteristic breakdown strength e) of ANF/SK/BNNS‐NH_2_ composite film and NHN paper before and after thermal aging.

Thermal stability was regarded as one of the key factors influencing the application reliability of insulating films. Figure S17a,b (Supporting Information) showed the TGA and DTG curves of pure ANF films and ANF/SK/BNNS‐NH_2_ composite films under a nitrogen atmosphere. The initial mass loss of the ANF film occurred at ≈200 °C, corresponding to the evaporation of physically adsorbed water. Due to the excellent thermal stability of BNNS‐NH_2_, the residual yield of the composite films at 800 °C was proportional to the mass fraction of BNNS‐NH_2_. All composite films exhibited higher initial decomposition temperatures (*T*_d_, defined as 10 wt.% mass loss) and maximum decomposition temperatures (*T*_max_) compared to the pure ANF film. Specifically, the *T*_d_ of the ANF/SK/BNNS‐NH_2_ composite film containing 15 wt.% BNNS‐NH_2_ increased from 505.49 to 523.19 °C, while the *T*_max_ increased from 547.57 to 558.06 °C. This improvement was attributed to the intrinsic thermal stability of BNNS‐NH_2_ and the restricted molecular chain mobility induced by the robust “brick‐and‐mortar” structure.^[^ ^71^ ^]^ Differential scanning calorimetry (DSC) was employed to further investigate the crystallinity and thermal stability of the composite films. As shown in Figure S17c (Supporting Information), no endothermic peaks were observed below 400 °C, indicating that the glass transition temperatures (*T*_g_) of both the pure ANF film and the ANF composite films exceeded 400 °C. The incorporation of BNNS‐NH_2_ had a negligible influence on the melting behavior of the composite films, confirming their excellent thermal stability. The application potential of the composite films was further evaluated using infrared thermography. A 3 W light‐emitting diode (LED) chip was employed as a heat source and connected to the film surface using thermally conductive copper adhesive to simulate the heat dissipation process during device operation. As shown in **Figures** **7**a,b, when the temperature reached steady‐state after 100 s, the hotspot temperatures of the ANF/BNNS‐NH_2_ and ANF/SK/BNNS‐NH_2_ composite films were reduced by 9.1 and 11.7 °C, respectively, compared to the pure ANF film (80.7 °C). Among the films with identical BNNS‐NH_2_ loading (40 wt.%), the ANF/SK/BNNS‐NH_2_ composite film exhibited the lowest hotspot temperature, which was consistent with the results of finite element simulations. These findings demonstrated that the ANF/SK/BNNS‐NH_2_ composite films were capable of effective heat dissipation in electronic devices and possessed great potential for thermal management applications.

During prolonged operation at elevated temperatures, composite films inevitably underwent a series of physical and chemical changes that led to performance degradation. An accelerated thermal aging test was conducted to evaluate the thermal aging resistance of the heat‐resistant commercial NHN insulation paper and the ANF/SK/BNNS‐NH_2_ composite film. After aging at 130 °C for 30 days, the tensile strength of the ANF/SK/BNNS‐NH_2_ composite film remained significantly higher than that of the NHN paper, both before and after thermal aging. Moreover, the tensile strength of the ANF/SK/BNNS‐NH_2_ film and the NHN paper decreased by only 1% and 2%, respectively, after aging (Figure 7c,d). The characteristic breakdown strength of the ANF/SK/BNNS‐NH_2_ film decreased from 371.63 to 350.57 kVmm^−1^ after aging, whereas that of the NHN insulation paper dropped from 174.96 to 164.63 kVmm^−1^, indicating a lower degradation extent for our composite film (Figure 7e). These results demonstrated that the thermal aging resistance of the ANF/SK/BNNS‐NH_2_ composite film reached the commercial standard and significantly outperformed the NHN insulation paper in terms of dielectric breakdown strength and mechanical properties, highlighting its broad application potential in high‐performance insulation systems.

## 3 Conclusion

In conclusion, this study developed a multifunctional ANF composite films by constructing a “rigid‐flexible” dual‐polymer network and introducing plasma‐functionalized BNNS‐NH_2_. The synergistic interactions between ANF, SK, and BNNS‐NH_2_ led to the formation of a robust 3D hydrogen‐bonding network, endowing the resulting composite film with excellent tensile strength (307.08 MPa), elongation at break (18.55%), and toughness (38.00 MJ m^−3^). The strong interfacial interaction with the polymer matrix enabled the full exploitation of the intrinsic thermal conductivity and insulating properties of BNNS‐NH_2_. Finite element simulations demonstrated that the substantially reduced interfacial thermal resistance significantly enhanced the thermal conductivity of the composite films (13.89 Wm^−1^K^−1^ at 40 wt.%). The dense and ordered biomimetic structure imparted the ANF/SK/BNNS‐NH_2_ composite films with remarkable thermal stability (*T*_d_> 520 °C, *T*_g_> 400 °C), low dielectric constant and loss, and outstanding thermal aging resistance. Moreover, the incorporation of an appropriate amount of BNNS‐NH_2_ effectively increased the trap depth, resulting in an ultrahigh breakdown strength (430 kVmm^−1^ at 10 wt.%), consistent with the finite element simulation results. These findings provide both theoretical and experimental insights for the design of high‐performance polymer‐based dielectric materials for advanced electrical and electronic applications.

## 4 Experimental Section

### Materials

Hexagonal Boron Nitride(h‐BN, 10–15 µm) was supplied by ENO Hi‐Tech Material Development Company, Qinhuangdao, China. SK(≈5 µm) powder was available from Dalian Meilun Biotechnology Co. in China. Kevlar 29 yarn was supplied by DuPont. Dimethyl Sulfoxide (DMSO), Acetone, Potassium Hydroxide (KOH), Lithium Citrate, Isopropyl Alcohol (IPA), Ethanol were supplied by Chengdu Cologne Chemical Reagent Co, Ltd, China.

### Preparation of BNNS‐NH_2_ and ANF

BNNS was prepared using ion intercalation‐assisted exfoliation (Figure S1, Supporting Information), followed by functionalization through plasma surface treatment. The dedicated RF (13.56 MHz) plasma system and the process of plasma modification of BNNS were shown Figure S2 (Supporting Information). The RF power supply (RSG 1000S, Changzhou Rexgel Electronic Technology Co., Ltd.) and the auto matcher (PSG‐IIAS, Changzhou Rexgel Electronic Technology Co., Ltd.) produced the RF power supply. A dielectric blocking discharge (DBD) structure was used to generate a high‐energy plasma in a vacuum atmosphere chamber, with a high‐voltage electrode diameter of 45 mm, a ground electrode diameter of 50 mm, a blocking medium of two high‐temperature‐resistant quartz glass sheets (100 mm × 100 mm × 1 mm), and an inter‐electrode distance of 3 mm. The plasma generator operated at a peak voltage of 9.5 kV, a frequency of 13.56 MHz, and a discharge power of 100 W. The entire process was conducted in a vacuum atmosphere chamber. The plasma treatment consisted of three steps, first, the BNNS was cleaned and surface activated with continuous wave argon plasma. Next, NH_3_ plasma was treated in pulsed mode for 2 min to achieve the provision of etching and the formation of amino functional groups on the BNNS surface. The first treatment step was immediately followed by the second step without removing the sample from the vacuum atmosphere chamber. Upon completion of the treatment, BNNS with surface‐grafted ‐NH_2_ was obtained, denoted as BNNS‐NH_2_.

ANF were prepared using a deprotonation method. First, Kevlar 29 yarns were cut into smaller pieces of less than 1 cm and then placed in acetone soaking and sonicated for 4 h to remove fiber surface impurities while loosening the fiber structure. It was subsequently washed repeatedly with ethanol and deionized water and placed under vacuum to dry at 45 °C for 48 h. Next, purified Kevlar 29 yarn (1.6 g) and KOH (2.4 g) were added to 500 mL of DMSO, and the obtained mixture was magnetically stirred (800 rpm) at 20 °C for 14 days, resulting in a dark red (3.2 mg mL^−1^) ANF/DMSO dispersion.

### Preparation of ANF/SK and ANF/SK/BNNS‐NH_2_ Composite Films

ANF/SK films were prepared using vacuum‐assisted filtration. First, a certain amount of SK powder (0, 2.5, 5, 7.5, and 10 mg) was diluted with 50 mL of DMSO solvent, and then added to the ANF/DMSO dispersion prepared in the previous step, and placed in the bath sonication for 3 h in order to form a homogeneous mixture of the dispersion. Subsequent addition of 4 % DI H_2_O was used to achieve protonation of the amide groups of the ANF, a step that is necessary for the formation of hydrogen bonds between the ANF and Fibroin. The prepared ANF/SK/DMSO dispersion was injected into 500 mL of DI H_2_O in small amounts in several times to generate ANF/SK flocculation, and then the ANF/SK gel was separated by vacuum filtration using a Buchner funnel to remove residual KOH and DMSO until the filtrate was pH 7. The gel dispersed in deionized water was subjected to vigorous shearing (14 000 rpm, IKA) to obtain a stable and homogeneous ANF/SK slurry, ultrasonicated to remove excess air bubbles in the slurry, and sequentially subjected to vacuum‐assisted filtration for 8 h on PTFE membranes (50 mm in diameter, pore size of 0.22 µm) to produce separate flexible ANF/SK film. All obtained films were placed between PTFE films and pressed at 15 MPa and 100 °C for 5 min (LP‐S‐50 ASTM, Labtech). For the construction of ANF/SK/BNNS‐NH_2_ composite films, first, BNNS‐NH_2_/DMSO and ANF/SK/DMSO dispersions were co‐mingled and then bath sonicated for 3 h, so that BNNS‐NH_2_ and ANF/SK were tightly connected, and then ANF/SK composite films were prepared using the same process as that of ANF/Silk.

### Characterizations

The microstructure and micromorphology of raw materials and composite films were characterized using a scanning electron microscope (SEM, ZEISS GeminiSEM 300, Germany) and a transmission electron microscope (TEM, Tecnai G2 F20 S‐TWIN). The thicknesses of BNNS and ANFs were tested by atomic force microscopy (AFM, Bruker Dimension ICON, Germany). X‐ray diffraction (XRD) was recorded on an X‐ray diffractometer (Rigaku Ultima IV, Japan) with a scanning rate of 5° min^−1^ and a 2θ range of 5 to 90°. X‐ray photoelectron spectroscopy (XPS, Kratos AXIS Ultra DLD spectrometer) measurements of BNNS using monochromatic Al Kα radiation. Fourier transform infrared (FT‐IR) spectra were recorded on a Nicolet is50 spectrometer (Thermo Fisher) in the wave number range of 400–4000 cm^−1^. The thermal stability properties of the BNNS and ANF films were analyzed using a thermogravimetric analyzer (TGA, Mettler TGA2, Switzerland) in an atmosphere of N_2_ (20 mL min^−1^) at a temperature range of 30–800 °C with a temperature increase rate of 10 °C min^−1^. Differential scanning calorimetry (DSC) analyses of pure ANF films and composite films were carried out on a TAQ2000 Differential Scanning Calorimeter with a temperature increase rate of 10 °C min^−1^ over the range of 30–400 °C and a nitrogen atmosphere (20 mL min^−1^). The thermal management properties of pure ANF films and composite films were characterized using infrared thermography (FLIR T650sc). The mechanical properties of the composite films were tested on a universal testing machine (119 INSTRON, USA) at a tensile rate of 1 mm min^−1^. Volume resistivity of films using Huace FE‐2000 Insulation Diagnostic Tester (Beijing Huace Testing Instrument Co., Ltd.). The dielectric breakdown strength of the composite films was measured using a dielectric breakdown testing device (DDJ50 kV, China) with a voltage ramp rate of 500 V s^−1^. The in‐plane thermal conductivity (*λ*) of the films was calculated using the equation:

(6) $$\lambda =\alpha \times C_{p}\times \rho$$

where *α* (mm^2^ s^−1^) is the rate of thermal diffusion in the plane measured at 25 °C using a NETZSCH LFA 467 laser flash tester; *C*_p_ (J g^−1^K^−1^) denotes the specific heat capacity of the composite film, which was measured using the DSC sapphire method; and *ρ* (g cm^−1^) denotes the density of the film, which was calculated from the mass (*m*) and the volume (*v*) by *ρ* = *m*/*v*.

## Conflict of Interest

The authors declare no conflict of interest.

## Supporting information



Supporting Information

## Data Availability

The data that support the findings of this study are available from the corresponding author upon reasonable request.

## References

[1] X. Jing , Y. Li , J. Zhu , L. Chang , S. Maganti , N. Naik , B. B. Xu , V. Murugadoss , M. Huang , Z. Guo , Adv. Compos. Hybrid Mater. 2022, 5, 1090.

[2] J. Han , G. Du , W. Gao , H. Bai , Adv. Funct. Mater. 2019, 29, 1900412.

[3] R. Roy , K. C. Stevens , K. A. Treaster , B. S. Sumerlin , A. J. H. Mcgaughey , J. A. Malen , A. M. Evans , Mater. Horiz. 2024, 11, 3267. 38747574 10.1039/d3mh01796f

[4] M. Zhang , W. Zhou , S. Zhao , J. Zhao , C. Li , J. Fan , Y. Jia , Inorg. Chem. Commun. 2025, 179, 114726.

[5] Y. Duan , H. Yu , F. Zhang , M. Qin , W. Feng , Nano Res. 2024, 17, 9796.

[6] R. Y. Tay , H. Li , H. Wang , J. Lin , Z. K. Ng , R. Shivakumar , A. Bolker , M. Shakerzadeh , S. H. Tsang , E. H. Teo , T. Nano Today 2023, 53, 102011.

[7] X. Yu , M. R. Bhatti , X. Ren , P. Steiner , F. Di Sacco , M. Dong , H. Zhang , D. Papageorgiou , G. Portale , C. Kocabas , C. W. M. Bastiaansen , M. Reece , H. Yan , E. Bilotti , Compos. Sci. Technol. 2022, 229, 109695.

[8] C. Guo , Y. Li , J. Xu , Q. Zhang , K. Wu , Q. Fu , Mater. Horiz. 2022, 9, 1690. 35393993 10.1039/d2mh00276k

[9] F. Kong , W. Zhou , F. Zhang , W. Li , H. Li , Y. Zhu , B. Zhou , T. Yao , B. Li , J. Mater. Chem. A 2025, 13, 8852.

[10] M. C. Vu , H. Kang , P. J. Park , B.‐G. Choi , J.‐W. Paik , W.‐K. Choi , M. A. Islam , Q. Wang , S.‐R. Kim , Chem. Eng. J. 2022, 444, 136504.

[11] X. Dong , B. Wan , J. W. Zha , Chem. Rev. 2024, 124, 7674. 38847509 10.1021/acs.chemrev.3c00802

[12] Y. Wu , L. Chen , Y. Han , P. Liu , H. Xu , G. Yu , Y. Wang , T. Wen , W. Ju , J. Gu , Nano Res. 2023, 16, 7801.

[13] F. Luo , W. Cui , Y. Zou , H. Li , Q. Qian , Q. Chen , Mater. Horiz. 2024, 14, 3386. 10.1039/d4mh00382a 38689529

[14] D. Li , E. Peng , F. Lu , B. Wang , Y. Shen , P. Liu , L. Liu , Y. Huang , Z. Hu , Chem. Eng. J. 2023, 455, 140887.

[15] J. W. Zha , F. Wang , B. Wan , Prog. Mater. Sci. 2025, 148, 101362.

[16] H. Zhai , J. Liu , Z. Liu , Y. Li , Adv. Fiber Mater. 2025, 7, 443.

[17] K. Dash , D. K. Panda , K. Yadav , S. Meher , M. Mishra , Appl. Nanosci. 2024, 14, 423.

[18] S. Zhang , Z. Shang , D. Ding , X. Wang , Y. Wu , S. Nian , Z. Liu , Q. Zhang , Y. Chen , IET Nanodielectr. 2024, 7, 150.

[19] L. Su , X. Ma , J. Zhou , X. Liu , F. Du , C. Teng , Nano Res. 2022, 15, 8648.

[20] D. Pan , J. Dong , G. Yang , F. Su , B. Chang , C. Liu , Y.‐C. Zhu , Z. Guo , Adv. Compos. Hybrid Mater. 2022, 5, 58.

[21] Y. Zhang , Y. Fan , U. Kamran , S.‐J. Park , Composites, Part A 2022, 156, 106869.

[22] Y. Yang , J. Chao , P. Jiang , R. Xu , Y. Li , Y. Zhan , Z. Shi , C. Liao , IET Nanodielectr. 2024, 7, 68.

[23] S. Wang , Q. Zhang , J. Tang , Y. Xu , M. Li , L. Feng , J. Xie , C. Wei , Y. Ding , Compos. Commun. 2022, 32, 101153.

[24] C. Martinez‐Jimenez , A. Chow , A. D. S. McWilliams , A. A. Marti , Nanoscale 2023, 42, 16836. 10.1039/d3nr03941b 37850487

[25] V. Vatanpour , S. A. Naziri Mehrabani , B. Keskin , N. Arabi , B. Zeytuncu , I. Koyuncu , Ind. Eng. Chem. Res. 2021, 37, 13391.

[26] X. Liu , Y. Gao , Y. Shang , X. Zhu , Z. Jiang , C. Zhou , J. Han , H. Zhang , Polymer 2020, 203, 122763.

[27] Z. Zheng , M. Cox , B. Li , J. Mater. Sci. 2018, 53, 66.

[28] N. Burger , A. L. , M. Ferriol , M. Lutz , V. Toniazzo , D. Ruch , Prog. Polym. Sci. 2016, 61, 1.

[29] S. Sabrin , S.‐H. Hong , S. Kumar KC , J.‐S. Oh , A. L. K. Derrick‐Roberts , D. K. Karmokar , H. Habibullah , R. D. Short , B. Ghimire , R. Fitridge , E. J. Szili , Adv. Funct. Mater. 2024, 34, 2314345.

[30] S. Tian , W. Chen , R. Wang , C. Qin , Z.‐J. Jiang , Z. Jiang , Adv. Funct. Mater. 2024, 34, 2408035.

[31] Y. Liu , Z. Jiang , Z.‐J. Jiang , Adv. Funct. Mater. 2023, 33, 2302883.

[32] J. Qiu , G. Chen , Y. Guo , T. Li , P. Tang , B. He , X. Zhang , J. Huang , Adv. Funct. Mater. 2025, 2508158.

[33] Y. Yin , B. Luo , K. Li , B. M. Moskowitz , B. M. Lis , I. E. Wachs , M. Zhu , Y. Sun , T. Zhu , X. Li , Nat. Commun. 2024, 15, 3592. 38678057 10.1038/s41467-024-47878-1 PMC11055856

[34] M. Di‐Oliveira , R. G. Rocha , M. C. Marra , T. C. Oliveira , M. Vojs , M. Marton , R. D. Crapnell , C. E. Banks , E. M. Richter , R. A. A. Muñoz , Electrochim. Acta 2025, 537, 146851.

[35] Z. Yang , X. Huang , J. Li , B. Tang , G. Huang , W. Wei , G. Wu , Compos. Interfaces 2023, 30, 1411.

[36] X. Meng , W. Zhou , X. Chen , F. Kong , J. Zhao , W. Li , Y. Zhang , F. Wang , M. Yuan , Mater. Today Chem. 2025, 43, 102492.

[37] Z. Wang , R. Wang , M. Luo , X. Cao , J. Wang , X. Tian , L. Li , Small 2025, 21, 2502696. 10.1002/smll.202502696 40227152

[38] M. Luo , Y. Zhou , R. Wang , X. Cao , Z. Wang , Chem. Eng. J. 2024, 501, 157623.

[39] G. Xiao , H. Li , Z. Yu , H. Niu , Y. Yao , Nano‐Micro Lett. 2024, 16. 10.1007/s40820-023-01252-w PMC10656391 37975956

[40] J. Wang , T. Song , W. Ming , M. Yele , L. Chen , H. Zhang , X. Zhang , B. Liang , G. Wang , Nano Res. 2024, 17, 2061. 10.1007/s40820-024-01496-0 PMC11469983 39392512

[41] Y. Guo , S. Wang , H. Zhang , H. Guo , M. He , K. Ruan , Z. Yu , G. Wang , H. Qiu , J. Gu , Adv. Mater. 2024, 36, 2404648. 10.1002/adma.202404648 38970529

[42] R. Zhai , H. Li , X. Wang , J. Wang , Z. Li , X. Guo , R. Wang , Y. Liu , K. Chen , J. Yang , D. Yu , C. Teng , X. Ma , J. Alloys Compd. 2024, 992, 174600.

[43] Q.‐F. Guan , H.‐B. Yang , Z.‐M. Han , Z.‐C. Ling , S.‐H. Yu , Nat. Commun. 2020, 11, 5401. 33144561 10.1038/s41467-020-19174-1 PMC7642342

[44] Y. Han , K. Ruan , J. Gu , Nano Res. 2022, 15, 4747.

[45] H. Xiang , L. Gao , D. Shi , L. Jiao , B. Cheng , N. Deng , G. Li , W. Kang , Adv. Fiber Mater. 2024, 6, 883.

[46] X. Tan , Q. Peng , Z. Stempień , J. Saskova , M. Venkataraman , J. Wiener , J. Militky , Fibers Polym. 2024, 25, 4215.

[47] B. Zhou , J. Song , B. Wang , Y. Feng , C. Liu , C. Shen , Nano Res. 2022, 15, 9520.

[48] Z. Zhang , Y. Wang , H. Zhou , H. Dai , J. Luo , Y. Chen , Z. Li , M. Li , C. Li , E. Gao , K. Jiao , J. Zhang , Adv. Fiber Mater. 2025, 7, 774.

[49] X. Zheng , X. Ma , Z. Yuan , X. Zhang , R. Zhai , R. Duan , X. Song , C. Teng , Y. Zhou , L. Jiang , Adv. Funct. Mater. 2025, 35, 2504519.

[50] N. Wang , G. Yang , H. Wang , C. Yan , R. Sun , C.‐P. Wong , Mater. Today 2019, 27, 33.

[51] X. Zheng , H. Cong , T. Yang , K. Ji , C. Wang , M. Chen , Nanotechnology 2022, 33, 185602. 10.1088/1361-6528/ac4b7c 35030544

[52] H. Oh , J. Kim , Compos. Sci. Technol. 2019, 172, 153.

[53] S. Zhang , W. Chen , Y. Zhao , K. Yang , B. Du , L. Ding , W. Yang , S. Wu , Composites, Part B 2021, 223, 109106.

[54] M. Yang , K. Cao , L. Sui , Y. Qi , J. Zhu , A. Waas , E. M. Arruda , J. Kieffer , M. D. Thouless , N. A. Kotov , ACS Nano 2011, 5, 6945. 21800822 10.1021/nn2014003 PMC3214697

[55] L. Xu , X. Zhao , C. Xu , N. A. Kotov , Adv. Mater. 2018, 30, 1703343. 10.1002/adma.201703343 29134692

[56] J. Wang , X. Jin , C. Li , W. Wang , H. Wu , S. Guo , Chem. Eng. J. 2019, 370, 831.

[57] L. Zhou , Z. Yang , W. Luo , X. Han , S.‐H. Jang , J. Dai , B. Yang , L. Hu , ACS Appl. Mater. Interfaces 2016, 8, 28838. 27704759 10.1021/acsami.6b09471

[58] T. Ma , Y. Zhao , K. Ruan , X. Liu , J. Zhang , Y. Guo , X. Yang , J. Kong , J. Gu , ACS Appl. Mater. Interfaces 2020, 12, 1677. 31820630 10.1021/acsami.9b19844

[59] L. Zhao , C. Wei , J. Ren , Y. Li , J. Zheng , L. Jia , Z. Wang , S. Jia , Ind. Eng. Chem. Res. 2022, 61, 8881.

[60] L. Zhao , W. Wu , L. Jia , Z. Zhang , Z. Wang , X. Huang , W. Ning , J. Ren , Compos. Interfaces 2022, 29, 659.

[61] J. Ren , G. Jiang , Z. Wang , Q. Qing , F. Teng , Z. Jia , G. Wu , S. Jia , Adv. Compos. Hybrid Mater. 2024, 7, 5.

[62] M. Li , Y. Zhu , C. Teng , Compos. Commun. 2020, 21, 100370.

[63] L. Zhao , C. Liao , Y. Liu , X. Huang , W. Ning , Z. Wang , L. Jia , J. Ren , Compos. Interfaces 2022, 29, 447.

[64] Q. Yan , W. Dai , J. Gao , X. Tan , L. Lv , J. Ying , X. Lu , J. Lu , Y. Yao , Q. Wei , R. Sun , J. Yu , N. Jiang , D. Chen , C.‐P. Wong , R. Xiang , S. Maruyama , C.‐T. Lin , ACS Nano 2021, 15, 6489. 33734662 10.1021/acsnano.0c09229

[65] K. Sato , H. Horibe , T. Shirai , Y. Hotta , H. Nakano , H. Nagai , K. Mitsuishi , K. Watari , J. Mater. Chem. 2010, 14, 2749.

[66] W. Zhou , G. Cao , M. Yuan , S. Zhong , Y. Wang , X. Liu , D. Cao , W. Peng , J. Liu , G. Wang , Z. Dang , B. Li , Adv. Mater. 2023, 35, 2207829. 10.1002/adma.202207829 36349800

[67] G. Duan , F. Hu , Y. Liang , D. Lu , W. Shao , R. Xu , Y. Wang , Z. Hu , Fibers Polym. 2025, 26, 1381.

[68] J. Yuan , S. Yao , W. Li , A. Sylvestre , J. Bai , J. Phys. Chem. C 2014, 118, 22975.

[69] L. Shao , J. Li , Y. Guang , Y. Zhang , H. Zhang , X. Che , Y. Wang , Mater. Des. 2016, 99, 235.

[70] H. Yuan , Z. Wang , D. Lan , S. Zhang , Z. Zang , G. Jiang , H. Wei , Y. Zhang , J. Zheng , J. Ren , G. Wu , S. Jia , Adv. Compos. Hybrid Mater. 2025, 8, 61.

[71] S. Cui , N. Song , L. Shi , P. Ding , ACS Sustainable Chem. Eng. 2020, 8, 6363.

