# Supplementary material for: Plasma‐Modified Boron Nitride Nanosheets for High‐Performance Aramid‐Based Dielectric Films with Enhanced Multifunctionality
Source: Adv Sci (Weinh). 2025 Oct 24;13(1):e16944. doi: 10.1002/advs.202516944 (PMC12767117; doi:10.1002/advs.202516944)
Supplement: Supplementary file 1 — Supporting Information [file ADVS-13-e16944-s001.docx]

**Supporting Information**

**Plasma-Modified Boron Nitride Nanosheets for High-Performance Aramid-Based Dielectric Films with Enhanced Multifunctionality**

Zi Wang^1^, Chao Bian^1^, Jia-Cheng Zhang^1^, Meng Gao^2^, Ying-Ying Tong^1^, Jun-Xue Chen^1^, Lin Zhang^1^, Ran Zhuo^2^, Jun-Wen Ren^1,^*, Jun-Wei Zha^3,^*, Shen-Li Jia^1^

*^1^College of Electrical Engineering, Sichuan University, Chengdu 610065, P.R. China*

*^2^China Southern Power Grid CSG Electric Power Research Institute, Guangzhou 610213, P.R. China*

*^3^State Key Laboratory of Alternate Electrical Power System with Renewable Energy Sources, School of Electrical and Electronic Engineering, North China Electric Power University, Beijing 102206, P.R. China*

**** Corresponding author.***

E-mail: myboyryl@scu.edu.cn/jwren_scu@163.com (J. Ren); zhajw@ncepu.edu.cn (J. Zha)

**S1 Preparation of BNNS.**

**
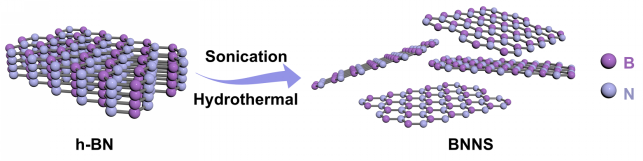
**

**Figure S1.** Schematic representation of the exfoliation process of BNNS.

BNNS was prepared by a combination of ion-assisted liquid phase stripping and hydrothermal methods (**Figure S1**). The h-BN powder (1.0 g) and lithium citrate (1.0 g) were first dispersed into 100 mL of deionised water/isopropanol (DI H_2_O/IPA, 3/1) mixed solvent, and the mixture was processed for 15 min under a tip sonicator (750 W, Ningbo Shanenzi Bio-technology Co., Ltd., China), followed by bath sonication (500 W, CHF-5B, Ningbo Shanenzi Bio Technology Co., Ltd. in China) for 6 h. The obtained dispersion was placed in a Teflon-lined stainless steel autoclave and hydrothermally treated at 200 °C for 6 h. Afterwards, the dispersion was centrifuged at 8000 rpm for 15 min to remove the supernatant, and the residual lithium citrate and isopropanol were further washed with DI water and filtered to remove the residual lithium citrate and isopropanol. After washing, it was re-dispersed in 150 mL of DI water and processed by bath acoustic treatment for 30 min, and then hydrothermally treated again in a Teflon lined stainless steel autoclave at 200 °C for 6 h to obtain an aqueous solution of BNNS, and then the obtained BNNS was dried in vacuum at 45 °C for 72 h. BNNS-NH2 was prepared after obtaining stripped good BNNS.

**S2 Schematic diagram and parameter configuration of the plasma treatment system.**


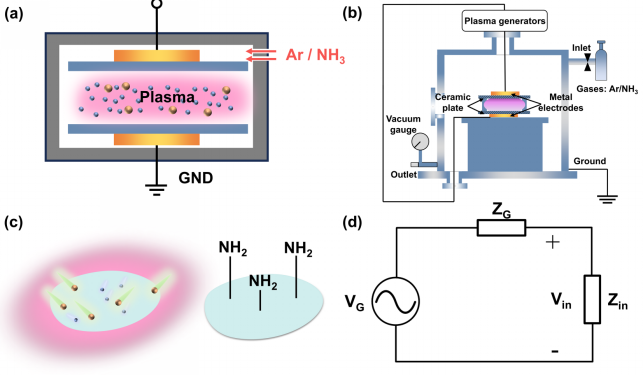


**Figure S2.** **(a)** and **(b)** Schematic diagrams of the plasma treatment system. **(c)** Diagrams showing the process of grafting amino groups on the surface of BNNS. **(d)** Impedance matching circuit schematic.

By connecting the power amplifier high voltage output to the resistance-capacitance matching box, automatic impedance matching mainly solved the conjugate matching problem between the RF power source and the plasma load. The maximum power was obtained when the load and the RF source are conjugate and equal. The circuit schematic was shown in **Figure S2d**. The electron energy distribution was optimised by adjusting parameters such as pulse duty cycle (PDC) and frequency. The calculation method and values for the PDC in this experiment were as follows:

$$PDC=t_{on}/\left(t_{on}+t_{off}\right)=1/5$$

Among these, *t*_on_ and *t*_off_ represent the plasma turn-on and turn-off times, respectively. Firstly, BNNS was exposed to continuous-wave argon plasma. This step effectively removed surface contaminants and weakly bound residues while introducing active surface sites through gentle etching. Next, NH_3_ plasma was introduced and ignited in pulsed mode Under vacuum conditions for 2 minutes. When a high-frequency electric field is applied to an NH_3_ molecule, the electrons in the NH_3_ molecule jump from a lower energy state to a higher energy state and the electrons are excited. When enough electrons are excited and there is sufficient energy, the NH3 molecule is ionised to form a plasma. Ammonia will ionise in a plasma cavity with the following ionisation equation:

$$NH_{3}+e^{-}\to NH_{2}+H+e^{-}$$

$$NH_{2}+e^{-}\to NH+H+e^{-}$$

$$NH+e^{-}\to N+H+e^{-}$$

These reactive species chemically interacted with the BNNS surface, simultaneously enabling mild surface etching and the grafting of amino functional groups (–NH_2_). The pulsed NH_3_ plasma thus provided strong and stable covalent anchoring sites while preserving the basal crystalline integrity of BNNS. After treatment, the BNNS was cooled to room temperature in situ under vacuum, preventing contamination from ambient gases. The resulting amino-functionalized nanosheets were denoted as BNNS-NH_2_.

**S3 Characterization of h-BN and BNNS.**


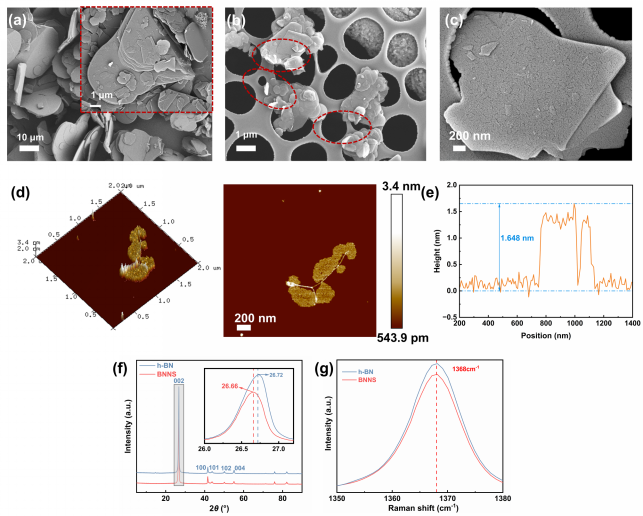


**Figure S3.** **(a)** SEM image of h-BN. **(b-c)** SEM images of BNNS. **(d, e)** AFM image of BNNS and the corresponding height profiles of BNNS. **(f)** XRD patterns of h-BN and BNNS. **(g)** Raman spectra for the h-BN and BNNS.

**S4 Characterization of BNNS and BNNS-NH_2_.**


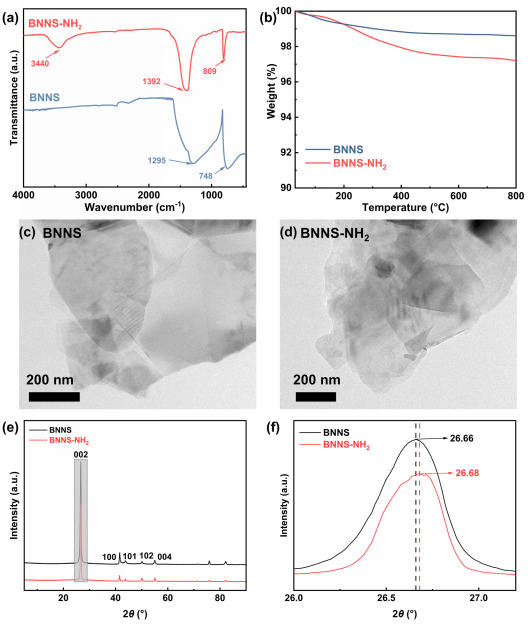


**Figure S4.** **(a)** FT-IR spectra of BNNS and BNNS-NH_2_. **(b)** TGA curves of BNNS and BNNS-NH_2_. TEM images of **(c)** BNNS and **(d)** BNNS-NH_2_. **(e, f)** XRD patterns of BNNS and BNNS-NH_2_.

**S5 Characterization of the dispersion properties of BNNS-NH_2_.**


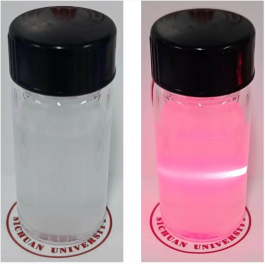


**Figure S5.** Schematic diagram of the dispersion of BNNS-NH₂ and the Tyndall effect.

**S6 Schematic of the preparation process of ANF.**


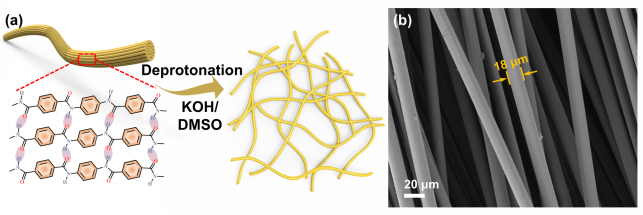


**Figure S6. (a)** Schematic diagram of the preparation of ANF. **(b)** SEM image of aramid fibers.

**S7 Characterization of mechanical properties of ANF/Silk Fibroin films.**


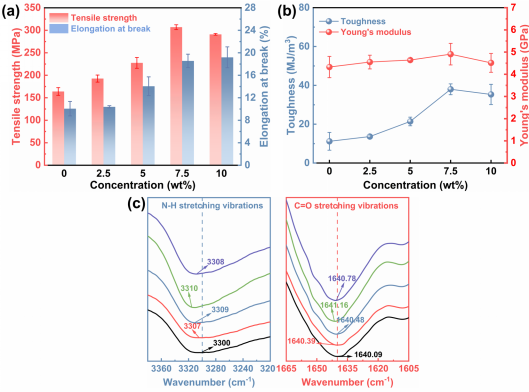


**Figure S7. (a)** Tensile strength and elongation at break of ANF/Silk Fibroin films with different Silk Fibroin concentrations. **(b)** Young's modulus and toughness of ANF/Silk Fibroin films with different Silk Fibroin concentrations. **(c)** FT-IR spectra of ANF/Silk Fibroin films with different Silk Fibroin concentrations.

**S8 Photographs and flexibility demonstration of ANF film and ANF/Silk Fibroin/BNNS-NH_2_ composite films.**


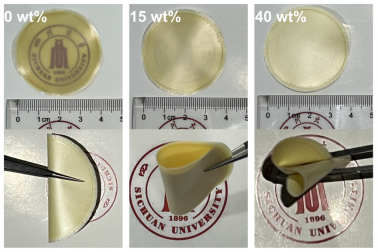


**Figure S8.** Photographs of ANF/Silk Fibroin/BNNS-NH_2_ composite films and their mechanical flexibility.

**S9 Characterization of the microstructure and mechanical properties of ANF/Silk Fibroin/BNNS-NH_2_ composite films**


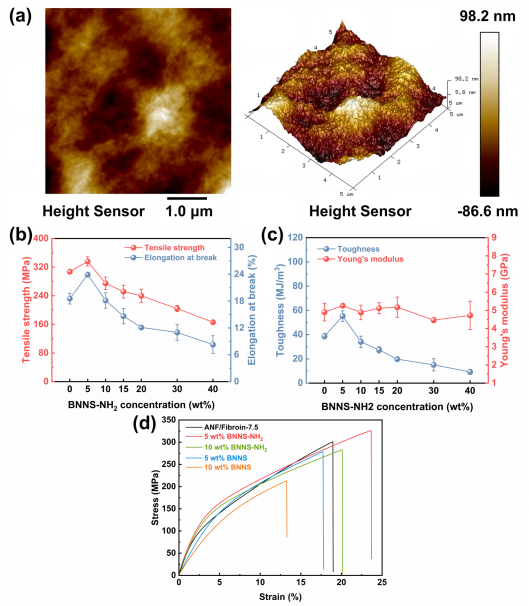


**Figure S9. (a)** AFM images of the surface morphology of ANF/Silk Fibroin/BNNS-NH_2_ composite film with 40 wt% BNNS-NH_2_ concentration. **(b)** Tensile strength and elongation at break of ANF/Silk Fibroin/BNNS-NH_2_ composite films with different BNNS-NH_2_ concentrations. **(c)** Young's modulus and toughness of ANF/Silk Fibroin/BNNS-NH_2_ composite films with different BNNS-NH_2_ concentrations.**(d)** Comparison of mechanical properties of different films before and after plasma treatment of BNNS.

**S10 Fitting of the interfacial thermal resistance in composite films.**

**
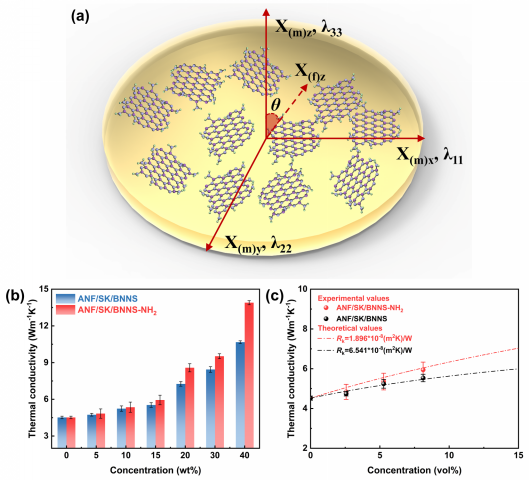
**

**Figure S10. (a)** Schematic diagram of in-plane and out-of-plane *λ* of the composite film. **(b)** Comparison of thermal conductivity of different films before and after plasma treatment of BNNS. **(c)** The experimental thermal conductivity of composite films and the theoretical values simulated the MG-EMT models.

The governing equation of the MG-EMT model that considers both the geometry and distribution characteristics of the two-dimensional fillers can be expressed as [S1]:

$$\lambda_{11}=\lambda_{22}=\lambda_{m}\frac{2+f_{BN}\left[\beta_{11}\left(1-L_{11}\right)\left(1+\left\langle\cos^{2}\theta\right\rangle\right)+\beta_{33}\left(1-L_{33}\right)\left(1-\left\langle\cos^{2}\theta\right\rangle\right)\right]}{2-f_{BN}\left[\beta_{11}L_{11}\left(1+\left\langle\cos^{2}\theta\right\rangle\right)+\beta_{33}L_{33}\left(1-\left\langle\cos^{2}\theta\right\rangle\right)\right]}$$

$$\lambda_{33}=\lambda_{m}\frac{1+f_{BN}\left[\beta_{11}\left(1-L_{11}\right)\left(1-\left\langle\cos^{2}\theta\right\rangle\right)+\beta_{33}\left(1-L_{33}\right)\left\langle\cos^{2}\theta\right\rangle\right]}{1-f_{BN}\left[\beta_{11}L_{11}\left(1-\left\langle\cos^{2}\theta\right\rangle\right)+\beta_{33}L_{33}\left\langle\cos^{2}\theta\right\rangle\right]}$$

with:

$$\beta_{\text{ii}}=\frac{\lambda_{ii}^{c}-\lambda_{\text{m}}}{\lambda_{\text{m}}+L_{\text{ii}}\left(\lambda_{ii}^{c}-\lambda_{\text{m}}\right)}$$

$$\lambda_{ii}^{c}=\frac{\lambda_{\text{BN}}}{1+\gamma L_{\text{ii}}\lambda_{\text{BN}}/\lambda_{m}}$$

$$\gamma=\left\{\begin{matrix} (2+1/p)\alpha, & \text{ for }p\geq1 \\ (1+2p)\alpha, & \text{ for }p\leq1 \end{matrix}\right.$$

$$\alpha=R_{\text{k}}\lambda_{\text{m}}/d$$

$$\left\langle\cos^{2}\theta\right\rangle=\frac{\int\rho(\theta)\cos^{2}\theta\sin\theta d\theta}{\int\rho(\theta)\sin\theta d\theta}$$

Here, *θ* is defined as the angle between the normal of the filler and that of the film (Figure S10). *L*_ii_ represents the depolarization factor, which satisfies:

$$L_{11}+L_{22}+L_{33}=1$$

$$L_{11}=L_{22}=\frac{p^{2}}{2\left(p^{2}-1\right)}+\frac{p}{2\left(1-p^{2}\right)^{1.5}}\cos^{-1}p$$

$$p=d/L$$

For fillers with a typical large aspect ratio such as BNNS-NH_2_ (*L* ≫ *d*), the condition p → 0 holds. Taking the limits of equations SXX and SXX yields: *L*_11_ = *L*_22_ = 0, *L*_33_ = 1, and *γ* = *α* = *R*ₖ*λ*ₘ/*d*. Substituting these values into the above equations and simplifying gives:

$$\lambda_{\text{c}}=\lambda_{\text{m}}\frac{2+f_{\text{BN}}\beta_{11}\left(1+\left\langle\cos^{2}\theta\right\rangle\right)}{2-f_{\text{BN}}\beta_{33}\left(1-\left\langle\cos^{2}\theta\right\rangle\right)}$$

$$\beta_{11}=\beta_{22}=\frac{\lambda_{\text{BN}}-\lambda_{\text{m}}}{\lambda_{\text{m}}}$$

$$\beta_{33}=1-\lambda_{\text{m}}\frac{1+\gamma\lambda_{\text{BN}}/\lambda_{\text{m}}}{\lambda_{\text{BN}}}$$

The Herman orientation factor *f* is a characteristic parameter used to evaluate the orientation state of two-dimensional fillers within the matrix, defined as:

$$f=\frac{3\left\langle\cos^{2}\theta\right\rangle-1}{2}$$

For a random orientation, *f* = 0, whereas *f* = 1 corresponds to fillers perfectly aligned in the horizontal direction. In the modified fitting, we introduced the Herman orientation factor as a free parameter, with its range set between [0.94, 1.00], representing the near-horizontal alignment of BNNS-NH_2_. The final governing equation of the modified MG-EMT model derived accordingly is:

$$\lambda=\lambda_{\text{m}}\frac{2+f_{\text{BN}}\left(\lambda_{\text{BN}}-\lambda_{\text{m}}\right)\left(1+\left\langle\cos^{2}\theta\right\rangle\right)/\lambda_{\text{m}}}{2-f_{\text{BN}}\left[(1-\gamma)\lambda_{\text{BN}}-\lambda_{\text{m}}\right]\left(1-\left\langle\cos^{2}\theta\right\rangle\right)/\lambda_{\text{BN}}}$$

For the high-loading regime, the Bruggeman model was applied for fitting, and the governing equation is given as follows[S2, S3]:

$$f_{BN}\frac{\lambda_{BN}-\lambda}{\lambda_{BN}+2\lambda}+\left(1-f_{BN}\right)\frac{\lambda_{\text{m}}-\lambda}{\lambda_{\text{m}}+2\lambda}=0$$

Considering the anisotropy of the thermal conductivity of the composite films, the Bruggeman model can be expressed in component form:

$$\sum_{i} f_{i}\frac{\lambda_{i}-\lambda_{j}}{\lambda_{i}+L_{j}\left(\lambda_{i}-\lambda_{j}\right)}=0$$

where *λ_i_* is the thermal conductivity of the filler, *λ_j_* is the effective thermal conductivity of the film along different directions of the Cartesian coordinate system, and *L* is the depolarization factor, which also satisfies equations S11-S13. By introducing the Herman orientation factor *f*, the following equation is obtained for the in-plane direction:

$$f_{BN}\frac{\lambda_{BN}^{*}-\lambda}{\lambda+L_{11}^{\text{eff}}\left(\lambda_{BN}^{*}-\lambda\right)}+(1-f_{BN})\frac{\lambda_{m}-\lambda}{\lambda+L_{11}^{\text{eff}}\left(\lambda_{m}-\lambda\right)}=0$$

$$L_{11}^{\text{eff}}=L_{33}\frac{1-f}{3}+L_{11}\frac{2+f}{3}=\frac{1-f}{3}$$

$$\lambda_{BN}^{*}=\frac{\lambda_{BN}}{1+2R_{k}\lambda_{BN}/d}$$

By treating the above equation as the expression for the in-plane thermal conductivity *λ* of the film, it can be rearranged into the standard quadratic form:

$$(2+f)\lambda^{2}+\left[2(f-1)\lambda_{m}+(1-4f)\lambda_{BN}^{*}\right]\lambda-(1-f)\lambda_{m}\lambda_{BN}^{*}=0$$

The volume fraction of the filler was calculated using the following equation:

$$f_{BN}=\frac{wt_{BN}/\rho_{BN}}{wt_{BN}/\rho_{BN}+\left(1-wt_{BN}\right)/\rho_{ANF}}$$

The value of *ρ*_BN_ was obtained from the product datasheet, while the density of *ρ*_ANF_ was measured using the displacement method.

**S11 Thermal field finite element simulation of composite films.**


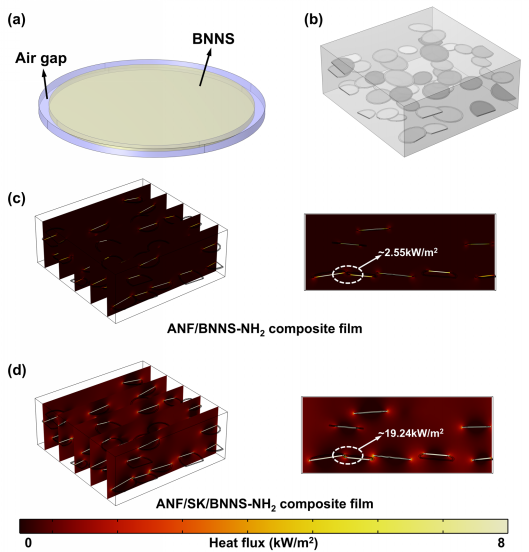


**Figure S11. (a, b)** Schematic diagram of finite element simulation modeling of the temperature field of composite film. Cross-sectional diagrams of heat flux distribution along the heat flow direction for **(b)** ANF/BNNS-NH_2_ composite film and **(c)** ANF/Silk Fibroin/BNNS-NH_2_ composite film.

The finite element simulation analysis was used to further investigate the contribution of interface interactions to thermal conductivity enhancement, which were implemented by COMSOL Multiphysics 6.1. Weak interface interactions were simulated by introducing air gaps around the fillers, whereas strong interface interactions were simulated without air gaps (**Figure S10a**). The process of constructing the simulation model was shown in **Figure S10b**, where a cuboid box was set up as the material matrix of ANF films with a measured *λ* of 4.52 Wm^-1^K^-1^. The disks represented BNNS and BNNS-NH_2_ with a *λ* of 390 Wm^-1^K^-1^. Notably, no air gaps were observed around BNNS-NH_2_, indicating a strong interfacial bonding with the matrix. Nearly horizontally aligned disks were randomly placed inside the cuboid box by the Monte Carlo algorithm. The left surface of the box was configured as a boundary heat source (500 W/m^2^). The right surface was set as a natural convection heat dissipation plane, and its heat flux density could be calculated from the natural convection equation (Newton’s law of cooling).

$$-n\left(-\lambda\nabla T\right)=h\left(T_{ext}-T\right)$$

The rest surfaces of the simulation box were assumed to be thermally insulating, and the boundary constraint equation was.

$$-n\left(\lambda\nabla T\right)=0$$

where *T* is the temperature of the cuboid, *T*_ext_ is the external ambient temperature, and *h* is the convective heat transfer coefficient. External ambient temperature was set as room temperature. A steady-state solver was used to calculate the entire heat transfer process. The heat diffusion equation is as follows.

$$\rho C_{p}u\nabla T+\nabla\left(-\lambda\nabla T\right)=Q$$

Where ρ is the density, *C*_p_ is the specific thermal capacity, and *Q* is the heat source.

**Table S1 Comparison of the *λ* and mechanical properties of composite films with other reported studies.**

| Polymer matrix | Fillers | *λ*  (Wm^-1^K^-1^) | Filler concentration (wt%) | Maximum Tensile strength (MPa) | Ref. |
| --- | --- | --- | --- | --- | --- |
| ANF | BNNS-NH_2_ | 4.52 | 0 | 307 | This work |
|  |  | 4.83 | 5 | 335 | This work |
|  |  | 8.58 | 20 | 239 | This work |
|  |  | 13.89 | 40 | 166 | This work |
| CNF | BNNS | 7.51 | 70 | 160 | [S4] |
| ANF | BNNS@PDA | 3.94 | 50 | 93.5 | [S5] |
| ANF | BNNS-OH | 8.96 | 20 | 275 | [S6] |
| ANF | F-graphene | 7.51 | 10 | 200 | [S7] |
| ANF | BNNS@PVP | 14.5 | 40 | 184 | [S8] |
| PI | h-BN | 7 | 60 | / | [S9] |
| ANF/AP | BNNS | 4.34 | 20 | 64 | [S10] |
| ANF | BNNS@TA@Ag | 12 | 40 | 101 | [S11] |
| PMMA | BN | 3.73 | 70 | / | [S12] |

**S12 AC conductivity of composite films.**


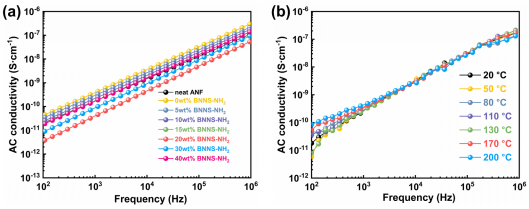


**Figure S12. (a)** Frequency dependence of AC conductivity for composite films with different BNNS-NH_2_ concentrations. **(b)** Frequency dependence of AC conductivity of ANF/Silk Fibroin/BNNS-NH_2_ composite films containing 15 wt % BNNS-NH_2_ at different temperatures.

**S13 Microstructure of fracture holes in ANF film and ANF/Silk Fibroin/BNNS-NH_2_ composite film.**


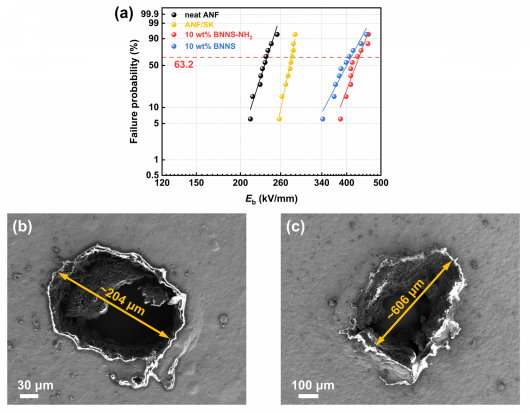


**Figure S13.** **(a)** Comparison of dielectric breakdown strength of different films before and after plasma treatment of BNNS. SEM images of fracture holes in **(b)** pure ANF film and **(c)** ANF/Silk Fibroin/BNNS-NH_2_ composite film.

**S14 Fitting results of TSDC curves for composite films with different BNNS-NH_2_ concentrations.**


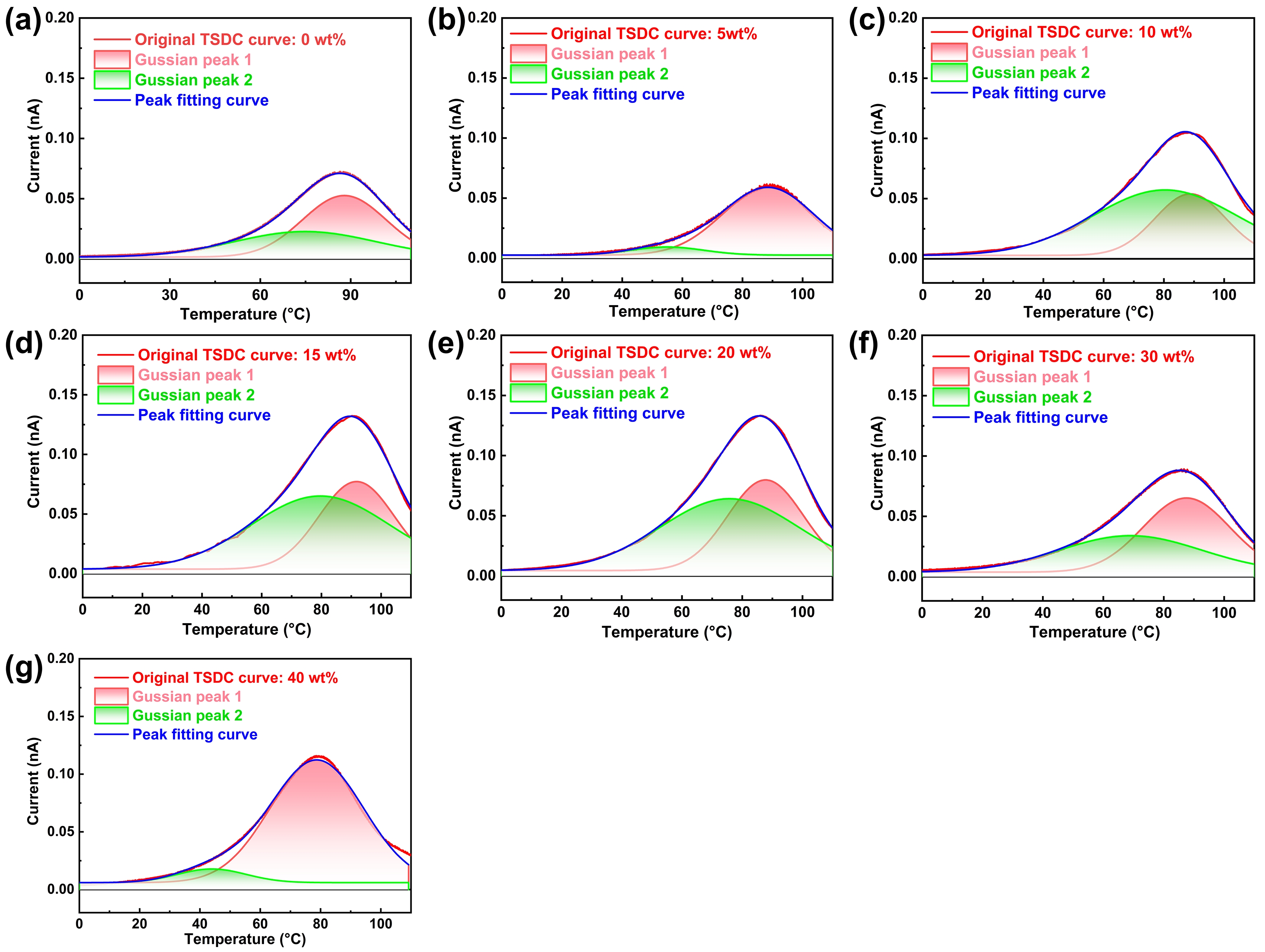


**Figure S14. (a-g)** Peak fitting of TSDC curves of composite films with different BNNS-NH_2_ concentrations.

**Table S2 Trap parameters of composite films with different BNNS-NH_2_ concentrations**

|  | E_1_(eV) | Q_1_(nC) | E_2_(eV) | Q_2_(nC) | R^2^ |
| --- | --- | --- | --- | --- | --- |
| 0 wt% | 0.820 | 37.2 | 0.464 | 26.6 | 0.999 |
| 5 wt% | 0.988 | 24.8 | 0.529 | 27.8 | 0.988 |
| 10 wt% | 1.009 | 35.2 | 0.470 | 64.4 | 0.998 |
| 15 wt% | 0.932 | 55.6 | 0.478 | 73.8 | 0.994 |
| 20 wt% | 0.837 | 51.4 | 0.483 | 73.0 | 0.999 |
| 30 wt% | 0.816 | 50.4 | 0.448 | 42.4 | 0.988 |
| 40 wt% | 0.722 | 34.4 | 0.821 | 76.4 | 0.996 |

**S15 Finite element simulation of the breakdown path development process in composite films.**


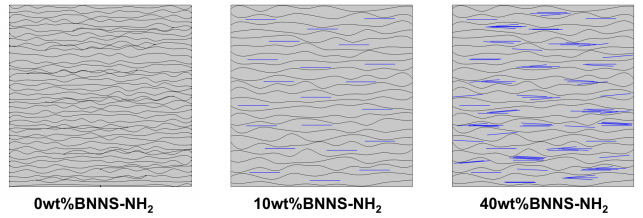


**Figure S15.** Schematic diagram of finite element simulation modeling of the electrical breakdown process of composite film.

Finite element analysis was employed to simulate the propagation behavior of the electrical breakdown path within the composite films. The physical field used in the model is the electrostatic field. The electric field distribution in the model was calculated by solving the Gaussian equation with the electric potential as the dependent variable. The relevant equation was given as:

$$-\nabla\cdot\left(\varepsilon_{0}\varepsilon_{r}\nabla T\right)=\rho_{v}$$

$$\nabla\cdot D=\rho_{v}$$

$$D=\varepsilon_{0}\varepsilon_{r}E$$

$$E=-\nabla V$$

where *ε*_0_ is the vacuum dielectric constant, *ε*_r_ is the relative dielectric constant, *V* is the electric potential, *ρ*_v_ is the electric charge density, *D* is the potential shift field, *E* is the electric field. The electrical breakdown model of the composite film was illustrated in **Figure S14**. A 2D simulation domain comprising a 240 × 240 grid was constructed. Boundary conditions were defined as follows: zero charge on the left and right edges, a fixed voltage applied to the top boundary, and the bottom edge grounded. The dielectric properties of the composite films were characterized by assigning relative permittivities of 1.5 for ANF and 3.9 for BNNS. In the framework of the fractal medium breakdown and percolation model, the evolution probability p of the electric tree channel was defined as follows:

$$p\left(i',j'-i,j\right)=\alpha+\beta-\gamma$$

$$\gamma=loss$$

where *ϕ_f_* is the threshold potential for electrical breakdown of the film, *ϕ_i_*_,_*_j_* , *ϕ_i’_*_,_*_j’_* and *ϕ_i”_*_,_*_j”_* are the potentials at the discharge point, the possible point and the link point, respectively, *η* is the fractal dimension, and *loss* denotes the evolutionary loss of the electric tree channel at the tip.

**S16 Aggregation of fillers inside composite films under high concentration.**


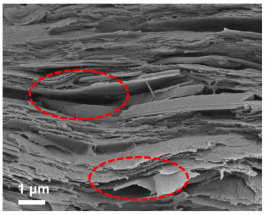


**Figure S16.** SEM image of typical agglomeration phenomena in composite film with high BNNS-NH_2_ concentration.

**S17 Thermal stability and accelerated thermal aging test of composite films.**


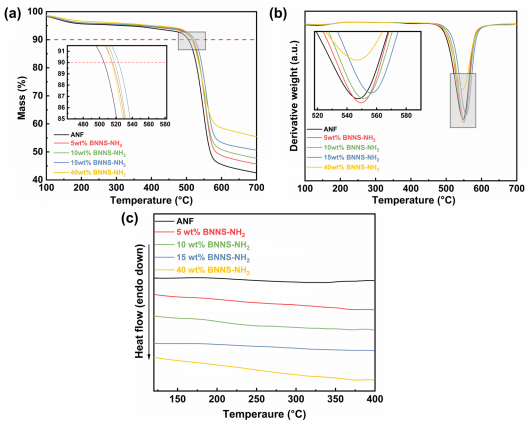


**Figure S17. (a)** TGA curves of pure ANF film and ANF/Silk Fibroin/BNNS- NH_2_ composite film. **(b)** DTG curves of pure ANF film and ANF/Silk Fibroin/BNNS-NH_2_ composite film. **(c)** DSC profiles of pure ANF film and ANF/Silk Fibroin/BNNS- NH_2_ composite membrane.

In this work, NHN refers to a laminated structure composed of a polyimide film sandwiched between two outer layers of Nomex® paper, which provides high thermal resistance and excellent insulating properties, and is widely used as a high-performance insulating medium in electrical and electronic equipment.

Accelerated thermal aging tests were used to investigate the thermal aging characteristics of composite films. The specific steps were as follows: Firstly, NHN insulating paper and the ANF/Silk Fibroin/BNNS-NH_2_ composite film were vacuum-dried at 45 ℃ for 48 hours. Subsequently, the samples were immersed in 50 mL of FR3 vegetable insulating oil (flash point: 360 ℃, Cargill) to simulate operational conditions. The treated samples were then subjected to an accelerated thermal aging test in a temperature-controlled chamber at 130 ℃ for 30 days.

Following aging, the mechanical and electrical properties of the samples were evaluated. According to the widely accepted 6 ℃ rule for thermal aging of transformer insulation systems-where the lifetime of Class A insulation is halved for every 6 ℃ increase above the benchmark temperature of 98 ℃-the equivalent lifetime loss of the insulation films after 30 days of aging at 130 ℃ was calculated using the following method:

$$30\times2^{\left(130-98\right)/6}=1209.5days\approx3.31years$$

Accordingly, thermal aging at 130 ℃ for 30 days resulted in an estimated equivalent service life loss of approximately 3.31 years.

Square specimens (10 × 10 mm^2^) of both ANF/SK/BNNS-NH_2_ films and commercial NHN paper were tested under identical conditions. All breakdown tests were conducted using cylindrical electrodes with a 3 mm diameter. The applied voltage was increased at a constant ramp rate of 500 V/s, and breakdown was defined when the leakage current reached 5 mA. Throughout the measurement, each specimen was fully immersed in transformer oil to suppress surface flashover and ensure a uniform electric field.

**References**

[S1] Nan, C.; Birringer, R.; Clarke D.; Gleiter H. Effective thermal conductivity of particulate composites with interfacial thermal resistance. *J. Appl. Phys.* **1997**, 81, 6692–6699.

[S2] Tahouni, S.; Azhdari, M.; Rezazadeh, G.; Fathalilou, M.; Pathak, R.; Ricken, T.; Seyedpour, S. Experimental and numerical analysis of heat transfer in polymercomposites with metallic inclusions using virtual element method. *Mater. Des.* **2025**, 255, 114172.

[S3] Bonfoh, N.; Jeancolas, A.; Dinzart, F.; Sabar, H.; Mihaluta, M. Effective thermal conductivity of composite ellipsoid assemblages withweakly conducting interfaces. *Compos. Struct.* **2018**, 202, 603–614.

[S4] Zhou, L.; Yang, Z.; Luo, W.; Han, X.; Jang, S.-H.; Dai, J.; Yang, B.; Hu, L. Thermally conductive, electrical insulating, optically transparent bi-layer nanopaper. *ACS Appl. Mater. Interfaces*. **2016**, *8*, 28838–28843.

[S5] Ma, T.; Zhao, Y.; Ruan, K.; Liu, X.; Zhang, J.; Guo, Y.; Yang, X.; Kong, J.; Gu, J. Highly thermal conductivities, excellent mechanical robustness and flexibility, and outstanding thermal stabilities of aramid nanofiber composite papers with nacre-mimetic layered structures. *ACS Appl. Mater. Interfaces*. **2020**, *12*, 1677–1686.

[S6] Zhao, L.; Wei, C.; Ren, J.; Li, Y.; Zheng, J.; Jia, L.; Wang, Z.; Jia, S. Biomimetic nacreous composite films toward multipurpose application structured by aramid nanofibers and edge-hydroxylated boron nitride nanosheets. *Ind. Eng. Chem. Res .***2022**, *61*, 8881–8894.

[S7] Zhao, L.; Wu, W.; Jia, L.; Zhang, Z.; Wang, Z.; Huang, X.; Ning, W.; Ren, J. Mechanically strong and thermally conductive paper made from aramid nanofiber and fluorinated graphene with excellent dielectric properties. *Compos. Interfaces*. **2022**, *29*, 659–673.

[S8] Ren, J.; Jiang, G.; Wang, Z.; Qing, Q.; Teng, F.; Jia, Z.; Wu, G.; Jia, S. Highly thermoconductive and mechanically robust boron nitride/aramid composite dielectric films from non-covalent interfacial engineering. *Adv Compos Hybrid Mater*. **2024**, *7*.

[S9] Sato, K.; Horibe, H.; Shirai, T.; Hotta, Y.; Nakano, H.; Nagai, H.; Mitsuishi, K.; Watari, K. Thermally conductive composite films of hexagonal boron nitride and polyimide with affinity-enhanced interfaces. *J. Mater. Chem.* **2010**, *20*, 2749.

[S10] Li, M.; Zhu, Y.; Teng, C. Facial fabrication of aramid composite insulating paper with high strength and good thermal conductivity. *Compos. Commun.* **2020**, *21*, 100370.

[S11] Zhao, L.; Liao, C.; Liu, Y.; Huang, X.; Ning, W.; Wang, Z.; Jia, L.; Ren, J. A combination of aramid nanofiber and silver nanoparticle decorated boron nitride for the preparation of a composite film with superior thermally conductive performance. *Compos. Interfaces*. **2022**, *29*, 447–463.

[S12] Oh, H.; Kim, J. Fabrication of polymethyl methacrylate composites with silanized boron nitride by in-situ polymerization for high thermal conductivity. *Compos Sci Technol.* **2019**, *172*, 153–162.
